# Markov chain Monte Carlo and expectation maximization approaches for estimation of haplotype frequencies for multiply infected human blood samples

**DOI:** 10.1186/s12936-016-1473-5

**Published:** 2016-08-25

**Authors:** Gie Ken-Dror, Ian M. Hastings

**Affiliations:** Liverpool School of Tropical Medicine, Pembroke Place, Liverpool, L5 3QA UK

**Keywords:** Haplotype reconstruction, Multiplicity of infection, Single nucleotide polymorphisms, Expectation–maximization algorithm, Markov chain Monte Carlo

## Abstract

**Background:**

Haplotypes are important in anti-malarial drug resistance because genes encoding drug resistance may accumulate mutations at several codons in the same gene, each mutation increasing the level of drug resistance and, possibly, reducing the metabolic costs of previous mutation. Patients often have two or more haplotypes in their blood sample which may make it impossible to identify exactly which haplotypes they carry, and hence to measure the type and frequency of resistant haplotypes in the malaria population.

**Results:**

This study presents two novel statistical methods expectation–maximization (EM) and Markov chain Monte Carlo (MCMC) algorithms to investigate this issue. The performance of the algorithms is evaluated on simulated datasets consisting of patient blood characterized by their multiplicity of infection (MOI) and malaria genotype. The datasets are generated using different resistance allele frequencies (RAF) at each single nucleotide polymorphisms (SNPs) and different limit of detection (LoD) of the SNPs and the MOI. The EM and the MCMC algorithm are validated and appear more accurate, faster and slightly less affected by LoD of the SNPs and the MOI compared to previous related statistical approaches.

**Conclusions:**

The EM and the MCMC algorithms perform well when analysing malaria genetic data obtained from infected human blood samples. The results are robust to genotyping errors caused by LoDs and function well even in the absence of MOI data on individual patients.

**Electronic supplementary material:**

The online version of this article (doi:10.1186/s12936-016-1473-5) contains supplementary material, which is available to authorized users.

## Background

Malaria infections in human blood often consist of several genetically-distinct infections, each of which is called a clone. Humans in endemic areas may receive up to 1000 infective bites per year. Polyclonal infections are common, the number of clones within a human blood sample called the multiplicity of infection (MOI). The average number of MOI is around three in humans who lives in areas of intense transmission, and rarely exceeds to 12 in any individual patient [1].

The presence of multiple clones (each of which is haploid) in a blood sample often makes it impossible to identify what multiple SNPs haplotypes are present in each patient. This makes estimating the frequencies of haplotypes in the malaria population from human blood samples a challenging computational task. Haplotypes are important in tracking anti-malarial drug resistance because genes encoding drug resistance may accumulate mutations at several codons in the same gene, each mutation increasing the level of drug resistance and possibly, reducing the metabolic costs of previous mutation. Drug resistance mutations threaten malaria control and treatment policies and the mutations and haplotypes that encode resistance will be the subject of this paper.

The prevalence of mutations (i.e. their presence/absence in a blood sample) can be directly observed so the information available for each human blood sample is (a) an estimate of the MOI and (b) the presence/absence of an allele at a SNP. In effect a blood sample provides a genotype whose ploidy level equals its MOI; the problem is to use these observed polyploid blood genotypes to infer haplotype frequencies. This inference is further complicated by genetic ambiguity that arises from three sources:

- The MOI is estimated using hyper-variable genetic loci, such as *msp1*, *msp2*, *glurp* and *ta109*, which typically have an expected homozygosity of around 0.05–0.08 [2]. Simple counting of the number of different alleles at each loci provides a minimum MOI. However, this may underestimate the population MOI if clones share alleles at hyper-variable loci purely by chance, or if they are low density clones missed during genotyping [3].
- Alleles at single nucleotide polymorphisms (SNPs) can only be scored as present/absent and not directly counted unless MOI ≤ 2. For example if MOI = 4 and both wildtype and mutant alleles are present in the sample, it is impossible to tell whether the ratio of mutant:wildtype clones is 1:3, 2:2 or 3:1.
- Differing assay sensitivity means that some alleles are not detected. Malaria clones in humans are not present at the same density. Differences arise because they are recognized differently by host immunity, and because of sequestration in their 48-h cycle in red blood cells. Alleles present in the numerically-smaller minor clones will provide fewer PCR amplification templates and hence a lower detection signal. The situation is further complicated by each allele’s signal strength being affected by other factors such as the size of the region amplified during PCR. Different laboratories set different cut-off levels to distinguish smaller true signals from background assay noise. The Swiss TPH attributed signals less than 30 % intensity of the main genotyping signals as ‘noise’, while other labs use lower cut-offs and some apparently rely on user subjectivity to distinguish minor peaks from technical noise. The cut-off defines as the assay’s limit of detection (LoD). If the LoD of PCR reactions differ between SNP and hyper-variable loci then it is possible to get the situation where an allele from a clone is detected at the hyper-variable locus used to determine MOI, but may be missed when genotyping the SNP at the resistance locus. Clones (usually at very low density) that are undetected at all loci can be ignored as they do not enter the analysis [1].

These three factors have a large impact when attempting to impute genetic data for the malaria population. In particular, it precludes estimating haplotype frequency by simple gene counting of unambiguous genotypes because low frequency alleles and haplotypes are systematically missed, typically leading to twofold errors in frequency estimates [1].

The impact of detection limits when genotyping blood samples are under-studied. A simulation developed to create artificial datasets that incorporate the three sources of genetic ambiguity described above. This allows for non-detection of clones and allows the user to know both the “true” underlying genetic data in the simulated dataset and the “observed” data that would be seen in the blood samples. This problem does not arise in conventional diploid species. They have equal copies of each chromosome so the genetic signal from each SNP allele is equal, hence the need to develop new ways of inferring haplotype frequency in malaria patients. Several statistical approaches to estimate haplotype frequencies from multiclonal infections have been proposed including: maximum-likelihood (ML) estimation using a hill climbing algorithm (MalHapFreq) [4], expectation–maximization (EM) using an efficient iterative maximum likelihood approach (malaria.em) [5] and a Metropolis–Hastings Markov Chain Monte Carlo implementation of a model constructed within a Bayesian framework, which we hereafter referred to as Bayesian [6]. The aim of the present study is to present two novel approaches i.e. MCMC and EM algorithms for haplotype reconstruction with known or unknown MOI, and to compare the results to those obtained from the related statistical approaches described above; In addition, quantify the impact of misclassified observed genotype and examine the accuracy of the various method in estimating the population haplotype frequency.

## Methods

The simulated datasets, estimation algorithms and statistical analysis described below have been implemented in the R statistical software system version 3.1.1 [7], on a 64-bit computer with 32.0 GB of random access memory and an Intel(R) Core(TM) i7-4770K central processing unit (CPU) @ 3.50 GHz processor.

### Simulation of genotype and haplotype datasets

#### Simulation of population (haplotype) data

The simulation starts by generating a user-defined number of human blood samples, N, (1, 2,…,N) in the dataset. The multiplicity of infection (MOI) in each blood sample is generated randomly by the default frequency distributions given by Jaki et al. [8] i.e. with “population” MOI frequencies as follows: = 1–4 %, 2–40 %, 3–10 %, 4–10 %, 5–20 %, 6–5 %, 7–6 %, 8–5 % [8]; this reflects a distribution of MOI observed in a relative intense area of malaria transmission.

Separate infections in the MOI are assumed to be genetically distinct and unrelated, haploid, asexual clones that are presumed to have been inoculated by separate mosquito bites into the same person. Each clone within the blood sample is then randomly assigned an allele from each of three hyper-variable genetic markers used to estimate its MOI. Here assume the loci *msp1*, *msp2* and *ta109* whose allele frequency distributions are given by Jaki et al. [8]. Each clone is then assigned a biomass randomly selected from the interval 10^9^ to 10^11^; the “biomass” is the total number of parasites in the human and this sampling interval is typical for symptomatic malaria infections. The relative biomass (i.e. its proportion of total biomass) of each clone is then calculated as that clone’s biomass divided by the total biomass in the patient. Importantly, the genotyping signal from a SNP or MOI allele will be assumed to be proportional to the relative biomass of parasites containing that allele.

Each clone is then assigned a resistance haplotype defined at a user-defined number of SNPs. This may be achieved using user-defined resistance allele frequencies (RAF) at each SNP in the haplotype and assuming linkage equilibrium (LE) between the codons. Alternately the haplotype frequencies can be input directly from user-defined haplotype frequencies if alleles at the SNPs are in linkage disequilibrium.

This approach was used to generate genetic datasets for subsequent analysis. Unless stated otherwise assumed: 100 blood samples per dataset, diallelic SNPs (i.e. either resistant or sensitive) RAF at each codon ranging from 1 to 50 %, and linkage equilibrium (LE) between all SNPs and MOI markers. 1000 datasets were generated and analysed assuming differing LoD i.e. 0.0/0.0, 0.1/0.05, 0.2/0.1, 0.3/0.15 where the first number is LoD_SNP_ and the second is LoD_MOI_.

#### Simulation of observed (genotype) data

Genotypes are the observable data obtained on human blood samples and are subjected to the sources of genetic ambiguity described above i.e. genotyping errors arising from LoD and the fact that different combination of haplotypes may give rise to the same observed genotype. The “true” genetic data are therefore processed as follows to simulate what would actually be observed in the blood samples.

*Observed MOI* The strength of each genotyping ‘signal’ is calculated from their relative biomasses. The cut-off for distinguishing true signals from ‘noise’ may differ slightly from that used for SNPs which is why having different detection limits for LoD_SNP_ and LoD_MOI_. The novel algorithm assumes a signal less than a certain proportion of the major signal, this proportion being denoted LoD_MOI_, is regarded as ‘noise’. So if LoD_MOI_ = 0.1, signals <10 % of the maximum would be regarded as noise and would not contribute to the ‘observed’ blood sample genotype. Alternative algorithms for distinguishing noise in MOI genotyping suggested (Additional file 1), and can be integrated into the code if required. The observed MOI is then calculated as the maximum number of the alleles observed at three hyper-variable genetic markers *msp1*, *msp2* and *ta109*.

*Observed genotypes* These are calculated in an analogous manner to MOI i.e. by assuming that a clone’s biomass determines its contribution to the genotyping signal. The total ‘signal’ for each allele at each SNP is then calculated and compared to the user-defined LoD_SNP_ to find which alleles are detectable and contribute to the observed blood genotype.

Finally run a “reality check” on the simulated blood dataset as would be done for real data. In particular, search for samples with observed MOI = 1 and one of the SNPs is heterozygous. These observations are incompatible and generally occur when MOI ≥ 2 but appears to have MOI = 1 for one of two main reasons. Firstly, the ≥2 clones are identical at all three MOI loci purely by chance such that the observed MOI = 1. Secondly, the clones do differ at one or more MOI loci, but difference in genotyping sensitivity (LoD) between MOI and SNPs means only a single MOI allele is detected at each hypervariable locus but a heterozygote is detected at one of the SNPs. In both cases, the MOI is reset to have a value of two as would likely occur when processing clinical samples.

### Novel haplotype reconstruction methods

#### The expectation–maximization (EM)-algorithm

Here after called the “EM” algorithm. This is a natural approach to estimating population parameters where the model depends on unknown latent variables [9, 10]. The EM-algorithm was first implemented for haplotype reconstruction by Excoffier and Slatkin [11], Hawley and Kidd [12], and Long et al. [13]. The EM method implemented here is a variation that incorporates MOI. It consists of several distinct steps and is explained in detail in the Additional file 1.

#### The Markov chain Monte Carlo (MCMC)-algorithm

Here after called the “MCMC” algorithm. This approach drawn iteratively samples in a way that each step the process should be drawing from a distribution that is becoming closer and closer to the target distribution [14–16]. The MCMC algorithm was first implemented for haplotype reconstruction by Stephens et al. [17], Stephens and Donnelly [18] and is implemented by us as described in the Additional file 1.

#### The confidence interval around haplotype frequency estimates

Once haplotype frequencies have been estimated, by either the EM or MCMC methods, the confidence interval (CI) around these estimates are calculated from the exact binomial tail areas [19] that are usually considered as the gold standard. The lower and upper bound of the interval are defined via quantiles of the *F* distribution:

1 $$\begin{aligned} &\frac{x}{{x + (n - x + 1)F_{2x,1 - \alpha /2}^{2n - 2x + 2} }} \\ & \quad \le \theta_{i} \le \frac{{(x + 1)F_{2n - 2x,1 - \alpha /2}^{2x + 2} }}{{x + (n - x + 1)F_{2n - 2x,1 - \alpha /2}^{2x + 2} }}\end{aligned} $$

where *x* = *θ*_*i*_*n*, *θ* is the haplotype frequency, *n* is number of blood sample (sample size) and *α* is the required width of the CI (so *α* = 0.95 for 95 % confidence intervals).

### Existing statistical methods of haplotype reconstruction

There are three other published methods that are available to use: malaria.em, Taylor et al. as R packages and MalHaploFreq software to infer haplotype frequencies that compared against the two novel methods described above.

- Maximum likelihood (ML) estimation using a hill climbing algorithm described in [4]. The approach was called MalHaploFreq and hereafter will be called the “MHF” algorithm. This algorithm uses a hill climbing as an iterative optimization method where the function to be maximized is evaluated at each step. The functions parameters are systematically varied each step with the goal to find a better solution than the previous one.
- Another expectation–maximization (EM) algorithm as described in [5]. Hereafter this will be called the “R-EM” approach. This is as efficient iterative maximum likelihood approach. The algorithm alternates between two steps expectation (*E*-step) the posterior probabilities of all haplotype combinations and maximization (*M*-step) the expectation of the log likelihood of the frequency estimates is maximized. The MOI values for each sample are used in the analysis if they are known. If unknown, MOI were assumed to follow a Poisson distribution with mean = 2. It differs from the EM method implemented here as the latter does not use the posterior probabilities of all haplotype combinations in the expectation.
- A Bayesian approach as described in [6]. Here after it will be called the “Bayesian” algorithm. It uses a Metropolis–Hastings Markov chain Monte Carlo (MH-MCMC), The MH-MCMC is used to draw samples of genotype frequencies conditional on the observed data. Each time a new genotype was sampled within the recursive re-sampling scheme. The genotype frequency samples drawn using the MCMC algorithm were then used to infer the relevant haplotype frequencies. The average of the frequency sample set was used as a point estimate of the haplotype frequencies. The algorithm starts with an initial estimate of haplotype frequencies, a vector of MOI in each patient, and a matrix of genotype counts. It proposes an update for the MOI vector and genotypes counts. The proposed MOI vector and genotype counts are accepted for rejected based on the Metropolis–Hastings ratio, which includes both the proposal densities and posterior densities. The MOI is based on prior distribution of four possible distributions (Uniform, Poisson, negative Binomial, and Geometric). The parameter of the distribution is set equal to the reported mean MOI. It differs to the MCMC method implemented here because, in its simplest form, the latter proposes an update only for the set of haplotypes because the MOI is known for each patient and the proposed set of haplotypes are accepted or rejected based on maximizing the conditional probability of observing the complete data.

### Evaluation of different statistical methods

There are several published methods and programmes for inferring malaria haplotype frequency [4–6], plus the MCMC and EM algorithm developed here, so objective metrics are required to quantify their relative performances. Simulate 1000 datasets as described above, assuming, for simplicity, that resistance is encoded at two loci (so there are four resistance haplotypes). Each dataset is obtained by a process of five sequential steps:

1. The population frequencies of haplotypes are defined by selecting a RAF for each locus at random and the four population haplotype frequencies obtained assuming linkage equilibrium between the alleles.
2. A field survey of malaria blood samples is simulated. Each patient in the dataset has an MOI assigned at random according to the frequencies given above. The malaria clones (a number equal to the MOI) are then sampled at random according to the “true” population frequencies of resistance haplotypes and polymorphic markers (*msp1*, *msp2*, *ta109*); note that the “*sampled*” resistance haplotype frequencies in the dataset will differ from the “true” frequency due to this sampling process.
3. The MOI polymorphic markers and resistance SNPs in each patient are then processed to obtain the ‘genotypes’ observed in the blood samples taken from patients (an example present in Table 1) depending on the LoD.
4. The “estimated” resistance haplotype frequencies are obtained from each of the statistical programmes described above.
5. Randomly select one “*estimated*” haplotype frequency from that dataset to evaluate the performance of the methods. One haplotype used in each dataset because the haplotype frequency estimates within each datasets are non-independent; for example a large deviation in estimating one frequency must be matched by a large error in another because the estimates must sum to unity.

**Table 1:** How malaria datasets are simulated

Patient #	MOI	BIOMASS	f.BIOMASS	*msp1*	*msp2*	*ta109*	Haplotype	Observed MOI	Observed genotype
*1*	1	5.29E+10	1.000	10	34	3	112	*1*	*112*
*2*	3	8.06E+09	0.100	24	23	5	112	1*	111*
		6.48E+10	0.803	20	6	5	111		
		7.86E+09	0.097	16	27	5	112		
*3*	2	5.06E+10	0.474	24	35	3	111	*2*	*111*
		5.62E+10	0.526	1	34	4	111		
*4*	2	5.52E+10	0.487	21	34	4	122	*2*	*133*
		5.81E+10	0.513	18	33	4	111		
*5*	3	3.16E+10	0.432	23	32	9	111	2*	133*
		1.35E+09	0.018	21	28	7	112		
		4.03E+10	0.550	23	27	9	122		

The ‘population’ frequencies of different MOI classes, polymorphic markers (*msp1*, *msp2*, *ta109*) and resistance haplotypes in the local malaria population are first defined. A number of patients are then simulated, five in this case but more usually 100. For each patient a MOI is first sampled according to the local “population” frequencies (which will depend on local transmission intensity). This MOI then determines the number of malaria clones in the patient. These clones are then simulated. The first step is to assign a biomass to the clone. The clone polymorphic markers are assigned at random according to the local true frequencies. Finally a resistance haplotype is assigned to the clone, again sampled from the local true frequencies. This process is repeated for each clone in each patient and gives rise to the data given in black font below. The genetic signal observed in each patient (last two columns) is then calculated as described in the main text. In this example, genetic signals are not detected if they constitute ≤10 % of the biomass (f.BIOMASS gives relative biomass for each clone in a patient). What is actually observed, and available for analysis, is the information given in italics; genotyping limits produce errors and those erroneous data are indicated by a asterisk: they are the data available to the researcher but do not truly reflect the genetic data of the parasites in that patient

*Haplotype* is the resistance haplotype for each clone. It is defined at three SNPs, for each clone: 1 = wildtype, 2 = mutat. *Observed genotype* is observed genotype for each patient. It is defined at three SNPs; for each SNP: 1 = wildtype alone, 2 = mutant alone, 3 = both wildtype and mutant genetic signals observed in the blood sample

Each of these five steps is repeated for each of the 1000 datasets. The datasets and selected haplotype in each dataset are kept the same for each of the five analysis method; this allows a direct comparison between the different methodologies used to infer haplotype frequencies.

The performance of the different methods is then measured as follows. Note that “population/sample” means that only one of these definitions should be used not that a division should be applied. These metrics are fairly standard ones used in haplotype reconstruction in conventional (i.e. diploid) organisms and details can be found elsewhere [11, 20–23]. Note that ‘*P*’ used below is a vector whose number of elements equal *h,* the number of potential haplotypes in the malaria population (in the sample case 2 resistance SNPs so *h* = 2^2^ = 4, but this may vary; for example if there are five SNPs then *h* = 2^5^ = 32 and so on). The elements of the vector are indicated by the superscript *i*.

### The accuracy of the estimates

The ‘population’ and “sampled” values are compared with the “estimated” value reported as:

The correlation coefficient *(R*^*2*^*)* between population/sample, and estimated haplotype frequency value is recorded for each of the 1000 selected haplotypes.

A similarity index (*I*_*F*_) [11] was calculated to examine how close the computationally estimated haplotype frequencies are to the ‘population’ and “sampled” haplotype frequencies as:

2 $$\begin{aligned} I_{F} &= \sum\limits_{i = 1}^{h} {\hbox{min} \left(Pi_{estimated} ,Pi_{population/sample}\right )} \\ &= 1 - \frac{1}{2}\sum\limits_{i = 1}^{h} {\left|Pi_{estimated} - Pi_{population/sample} \right|}  \end{aligned}$$

*P*_*estimated*_ and *P*_*population/sample*_ denote, respectively, the estimated and the population/sample haplotype frequency of *i* haplotype. This measure incorporates all *h* haplotype frequencies and thus captures the overall difference between *estimated* and *population/sample* frequencies. It varies between one, when *population/sample* and *estimated* haplotypes frequencies are identical, and zero, when *estimated* haplotypes frequencies tending to zero.

The mean squared error (*MSE*) [20] was calculated as:

3 $$ MSE = \frac{{\left[ {\sum\nolimits_{i = 1}^{h} {\left(Pi_{estimated} - Pi_{population/sample} \right)^{2} } } \right]}}{h} $$

*P*_*estimated*_ and *P*_*population/sample*_ denote, respectively, the *estimated* and the *population/sample* haplotype frequency of *i* haplotype, *h* is the number of haplotype frequencies in the population.

Since these indexes (*I*_*F*_, and *R*^*2*^) gives more weight to high frequency haplotypes the change coefficient *C* [21] assess the scaled change in haplotype frequencies and was calculated as:

4 $$ C_{i} = \frac{{\left(Pi_{estimated} - Pi_{population/sample} \right)}}{{Max\left[Pi_{estimated},\,Pi_{population/sample}\right ]}} $$

The coefficients were computed for each possible haplotype across statistical methods and presented as plot difference of estimation (%) on Y-axis against the haplotype frequency for each estimate on the X-axis. This metric is useful as it indicate that the haplotype frequency *estimated* and the haplotype frequency *population/sample* is the same. The value of the coefficient *C* ranges from 1 to −1, the value 0 indicating that the haplotype frequency estimated and the haplotype frequency population/sample are identical. Positive values indicate that haplotype frequency estimates tend to be larger than the *population/sample* frequency.

The *validity* of the methods measure how often the “population” and “sampled” frequencies fall within the 95 % confidence intervals (CI) of the estimated frequency. It would expect ~5 % of “population” values to fall outside the CI and ≤5 % of “sampled” values to fall outside the CI.

The *speed* of the analyses which is self explanatory recorded and presented as a line charts.

## Results

Three hyper-variable genetic markers *msp1*, *msp2* and *ta109* are used to estimate MOI. The observed MOI misclassifies (underestimates) population MOI by ~5 % even when the LoD_SNP_ and LoD_MOI_ are zero; this occurs when genetic profiles match purely by chance. Increasing LoD_MOI_ misclassifies the population MOI by increasing amounts i.e. by 8, 11 and 14 % when assuming LoD_(SNP/MOI)_ 10 %/5 %, 20 %/10 %, 30 %/15 %, respectively. Similarly errors arise when, increasing LoD_SNP_ as the observed genotypes are not necessary the true ones. Samples that are pure mutant or pure wildtype at SNPs will always be correctly classified (there are no minor genotyping signals at these SNPs) but genotypes at SNPs that are mixed mutant/wildtype may be misclassified as pure mutant or pure wildtype if the minor signal is lost. The mixed mutant/wildtype genotype was misclassified as pure mutant or pure wildtype by 5, 11 and 17 % among LoD_(SNP/MOI)_ 10 %/5 %, 20 %/10 %, 30 %/15 %, respectively (as expected the true and observed SNP genotypes are identical when the LoD_SNP_ are zero). These underestimated MOI values and misclassified genotypes caused by LoD potentially affect many of the subsequent estimates of haplotype frequencies as will be described below.

**Fig. 1:**
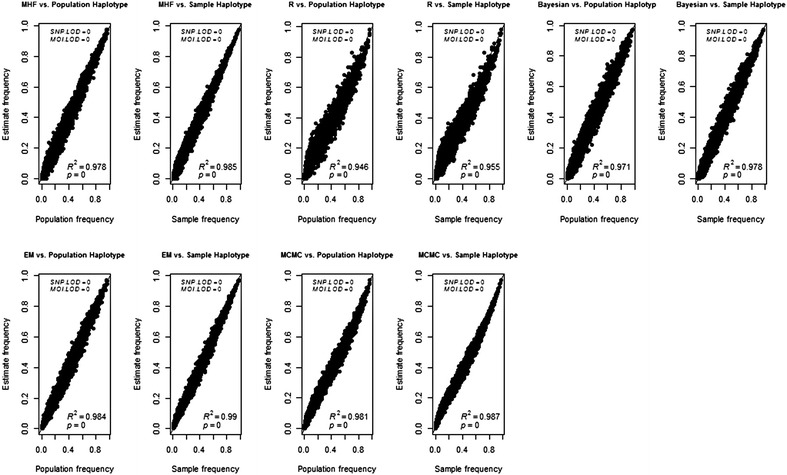
The correlation (*R* ^*2*^) between population/sample and estimated haplotype frequency across statistical methods among LoD_SNP/MOI_ = 0.00/0.00

The estimated haplotype frequency between the five statistical methods i.e. MHF (MalHaploFreq), R-EM (malaria EM), Bayesian (Bayesian statistic), EM (EM-algorithm), MCMC (Markov Chain Monte Carlo) and the population/sample haplotype frequencies among four combinations of LoD_(SNP/MOI)_ showed high concordance. Figure 1 shows the absolute deviation of the estimated haplotype frequency from population/sample haplotype frequency. The correlation coefficient (*R*^*2*^) is slightly higher by 0.49–0.85 % in the sample haplotype compared to the population haplotype among all statistical methods. Increasing both LoD_MOI_ and LoD_SNP_ decreases the correlation coefficient by 0.20–0.36 % among MHF, Bayesian and EM methods. Conversely, increasing both LoD_MOI_ and LoD_SNP_ increased the correlation coefficient by 3.06–3.29 % among R-EM method and by 0.20–0.15 % among MCMC method (Additional file 1: Figures S1–S3). The difference between correlation coefficients among statistical methods is less than 3.7 %. The data points lie close to the force line the diagonal through [0, 1]. There was also a tendency for the estimates to cluster more closely around the force line at high frequencies, showing that there is a tendency for high-frequency haplotypes to be more accurately estimated. Figure 1 reveals the presence and extent of bias as systematic deviations from the force line. The R-EM and MCMC methods both show some bias with high frequencies being slightly underestimated and low frequencies being slightly overestimated. However the bias is slight and changes with limits of detection. Additional file 1: Figures S1–S3 are analogous to Fig. 1 but illustrate the effect of increasing LoD. At higher limits (Additional file 1: Figure S3) the MHF and Bayesian methods appear to overestimate high frequency haplotype and under-estimate low frequency haplotypes.

**Fig. 2:**
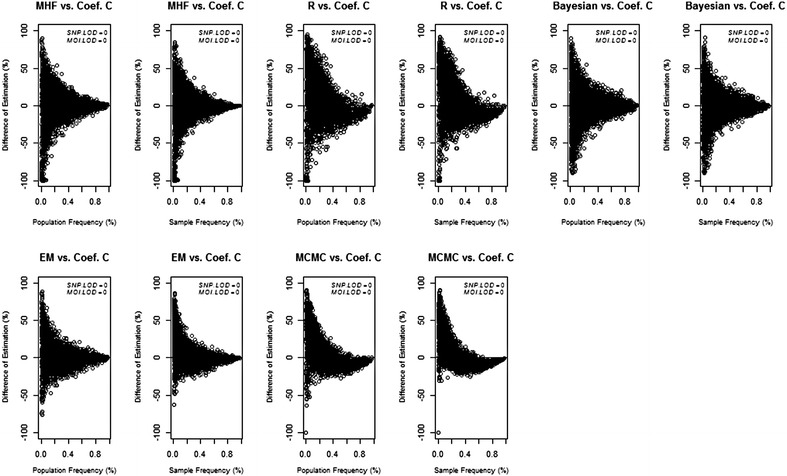
The change coefficient (*C*) between population/sample and estimated haplotype frequency across statistical methods among LoD_SNP/MOI_ = 0.00/0.00

The data on Fig. 1 can be processed to show the change coefficient *C* which incorporates both the direction and the percentage change between the estimated and population/sample frequencies (this deviation is known as “change” in the haplotype literature [21] although it would elsewhere generally be called “error”). In this literature a “small change” is conventionally denoted as *C* ≤ 0.15 [21]. Figure 2 demonstrates that dramatic values of *C* i.e. ≥90 % occur at the lowest haplotype frequencies. The worst estimates (*C* ≈ 1) occur when haplotype frequencies are less than around 7 % using MHF. In addition, poor estimates occurs at haplotype frequency <6 % among R-EM and Bayesian statistical methods. A single example of *C* ≈ 1 occurred at haplotype frequency 2.7 % among MCMC statistical methods. No such examples of *C* ≈ 1 occur at the EM statistical methods. Increasing both LoD_SNP_ and LoD_MOI_ (Additional file 1: Figures S4–S6) slightly decreases the lowest haplotype boundary at which *C* ≈ 1 occurs i.e. to <5 % among MHF, <4 % among R-EM and Bayesian statistical method, <2 % among EM statistical method and <1 % among MCMC statistical method. Approximately two-thirds (68 %) of the haplotype frequency estimates show either no change or small change (defined as *C* < 15 %) at LoD_SNP_ of 0.00 and LoD_MOI_ of 0.00 among MHF, EM statistical method and MCMC statistical method. On the other hand only 47 % among R-EM and 64 % among Bayesian statistical method of the haplotype frequency estimates show either no change or small change. Increasing both LoD_SNP_ and LoD_MOI_ to 0.30 and 0.15, respectively decreased the haplotype frequency estimates that show either no change or small change to 48 % among MHF, 49 % among Bayesian statistical method and 64 % among EM and MCMC statistical methods. On the other hand, increase the haplotype frequency estimates that show either no change or small change to 68 % among R-EM statistical method (Additional file 1: Figure S6). Figure 2 confirms the bias shown on Fig. 1, i.e. that R-EM and MCMC tend to underestimate high frequency haplotypes and over-estimate the low frequency ones.

**Fig. 3:**
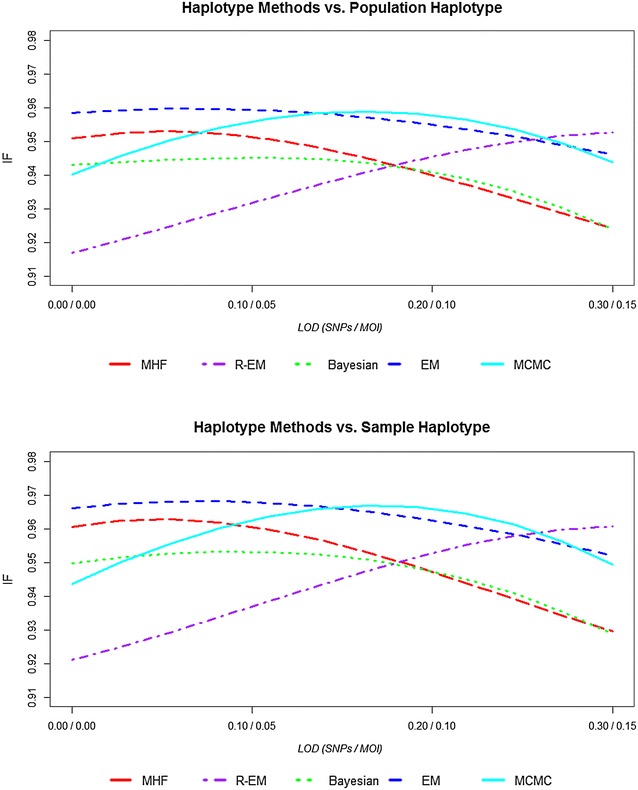
The similarity index of the estimates haplotype frequency compared population/sample haplotype frequency across statistical methods

**Fig. 4:**
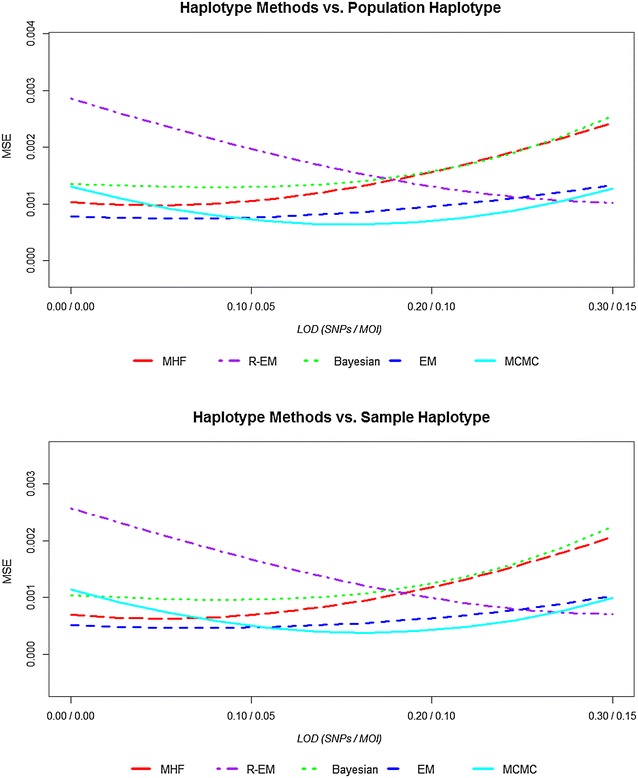
The *MSE* of the estimated haplotype frequencies compared to population/sample haplotype frequency across statistical methods

Figure 3 show the similarity index (*I*_*F*_) of the estimates haplotype frequency compared population/sample haplotype frequency. The five statistical methods provided similarity index (*I*_*F*_) values very close to each other. The similarity index is higher by 0.05–1.0 % in the sample haplotype compared to the population haplotype among all statistical methods and decreased with increasing LoD_SNP_ (30, 20, 10, 0 %) and LoD_MOI_ (15, 10, 5, 0 %) show in the Fig. 3. The difference between similarity indexes among statistical methods is less than 4 %. Increasing both the LoD_SNP_ and LoD_MOI_ decreases the similarity index between 2 and 3 % in MHF, and Bayesian methods, by 1 % in EM method; conversely, R-EM shows increasing values of *I*_*F*_ while the MCMC analyses is more complex, *I*_*F*_ increasing slightly then decreasing slightly. The similarity index gives more weight to common haplotypes whose frequencies are the most accurately estimated. This tendency is reflected in the *MSE* statistics shown. Figure 4 shows the mean squared error (*MSE*) of the estimated haplotype frequencies around the population/sample haplotype frequency. The *MSE* is lower by 0.0002–0.0004 in the sample haplotype compared to the population haplotype among all statistical methods. The difference between *MSE* between statistical methods is less than 0.002. Increasing both the LoD_SNP_ and LoD_MOI_ increased the *MSE* by 0.001 in MHF, and Bayesian methods, and slightly in EM methods (0.0005); conversely R-EM and MCMC methods decrease the *MSE* values by 0.002 and 0.0002, respectively.

**Fig. 5:**
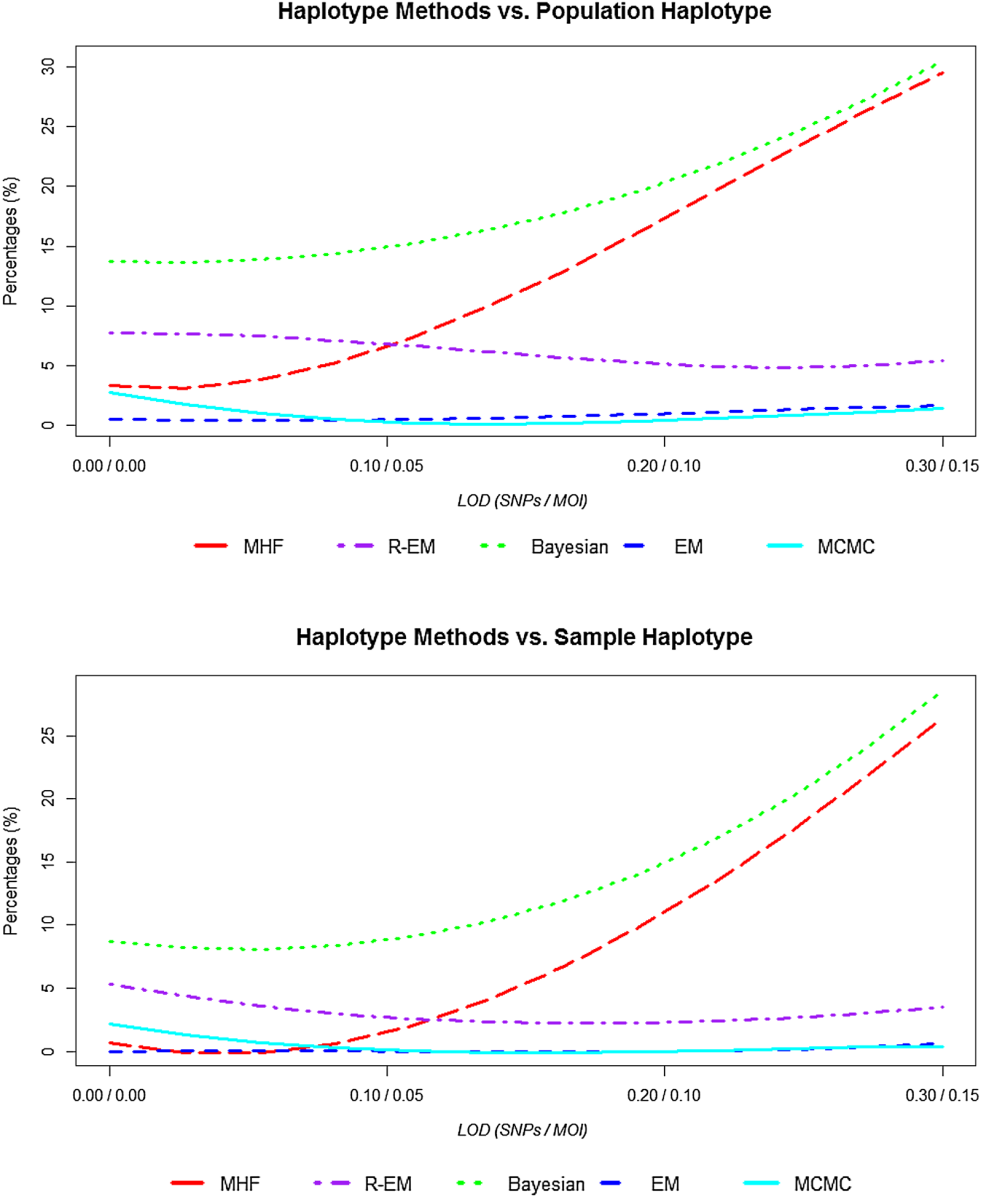
The validity of the methods, calculated “population/sampled” frequencies fall out of the 95 % CI

The validity of the methods can be quantifies as how often the “population” and “sampled” frequencies fall out of the 95 % confidence intervals (CI) generated by the analyses. It would expect ~5 % of “population” values to fall outside the CI and <5 % of “sampled” values to fall outside the CI. Figure 5 shows that, when LoD = 0 then the EM and MCMC methods all produce very narrow CI, while MHF is about correct (containing 95 % of the values) while the R-EM and Bayesian methods produces CI that are too wide with only about 85 and 90 % respectively of true values being contained within the CI. One explanation for the differences in performance is the way the CI was calculated. The MHF statistical methods calculate 95 % CI boundaries as occurring when the likelihood is less than 2 log units below the maximum-likelihood. The R-EM methods calculate 95 % CI from the standard error of the estimated haplotype frequencies. The Bayesian methods calculate 95 % CI as quintiles from haplotype frequency matrix. The EM and MCMC statistical methods calculate 95 % CI base on exact binomial tail areas (Eq. 1). A more fundamental difference between the methods is that Bayesian and R-EM did not incorporate MOI into the calculations; worryingly, both methods produced haplotype estimates that often lay outside the CI even when molecular detection was perfect. The percentage of results falling outside of the 95 % CI is slightly lower, by 2–5 %, in the sample haplotype frequency compared to the population haplotype frequency among MHF, R-EM, and Bayesian methods. Increasing both the LoD_SNP_ and LoD_MOI_ increased dramatically the error rates produced by Bayesian and MHF approaches with 27.4–26.3 %, and 15.6–17.4 % of estimates lying outside the 95 % CI among. EM methods were robust to changes in LoD with variation in the percentage falling outside the 95 % CI being <1.6 % across the four LoD assumptions. The R-EM and MCMC methods were similarly stable with variation being <2.1 and <2.8 % respectively. In summary, the difference between the missed CI between statistical methods is almost 30 %.

**Fig. 6:**
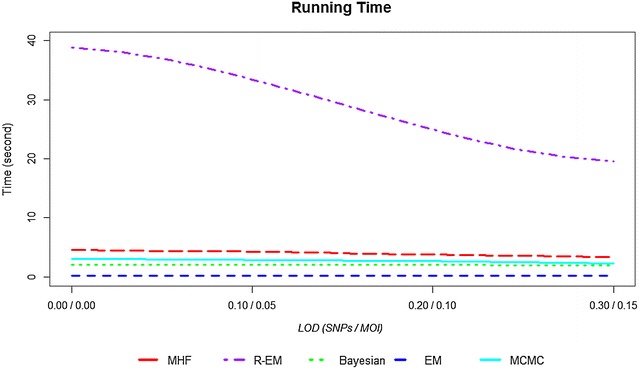
Computational time for five methods

Figure 6 shows the computational time for the statistical methods. There is a big difference between the statistical methods of almost 39 s. Increasing LoD_MOI_ and LoD_SNP_ decreased the time of the analysis by 74 % among MHF, 50 % among R-EM, 92 % among Bayesian, 77 % among EM, and 75 % among MCMC. The most likely explanation for the reduction in time taken to run the analyses is that as LoD increases, the observed MOI and genetic diversity within patients tends to decrease; consequently the datasets become slightly simpler and their analysis faster.

The haplotype frequency estimations for real data among five statistical methods are shown in Table 2. This was anonymized data kindly provided by colleagues at the Swiss Tropical and Public Health Institute; it has not yet been published so details cannot be provided here except to note that the data came from a sub-Saharan country in an area of intense transmission. The data set containing two biallelic SNPs for 82 individual with high MOI was used to check the applications of five statistical methods (MHF, R-EM, Bayesian, EM and MCMC). The results obtained from different methods were very similar with the mean difference of estimated haplotype frequencies between the statistical methods is about 3 %. The lowest difference of estimated probability is 1.5 % present between R-EM and MCMC methods and the highest difference of estimated probability is 6 % present between Bayesian and EM methods. These small differences were observed for all the data sets so only one set of results is shown.

**Table 2:** The haplotype frequency estimations for real data set (Swiss TPH), n = 82 individual

SNP 1	SNP 2	MHF	R-EM	Bayesian	EM	MCMC
1	1	0.928 (0.892–0.955)	0.914 (0.858–0.970)	0.936 (0.907–0.960)	0.908 (0.821–0.961)	0.914 (0.828–0.965)
1	2	0.011 (0.002–0.030)	0.016 (0.000–0.041)	0.017 (0.006–0.037)	0.013 (0.000–0.070)	0.021 (0.002–0.083)
2	1	0.004 (0.000–0.017)	0.005 (0.000–0.018)	0.007 (0.001–0.017)	0.009 (0.000–0.064)	0.008 (0.000–0.061)
2	2	0.057 (0.033–0.089)	0.065 (0.008–0.121)	0.038 (0.017–0.064)	0.069 (0.024–0.150)	0.058 (0.017–0.135)

*SNP* *1* = wildtype alone, *2* = mutant alone, *MHF* MalHaploFreq, *R-EM* malaria.em, *Bayesian* Bayesian statistic, *EM* EM algorithm, *MCMC* Markov chain Monte Carlo

## Discussion

This study proposed two statistical methods EM-algorithm and MCMC for haplotype reconstruction for multiply infected human blood samples. These methods have three major advantages over existing statistical methods: increased accuracy, validity (i.e. the percentage of true frequencies falling within the 95 % credibility limits) and their ability to return the probabilities of possible haplotype combination in each individual in the EM-algorithm or the uncertainly probability of the haplotype frequencies in the MCMC. Unfortunately, real data sets do not exist with known haplotypes to allow statistical comparisons of different methods. This study used simulation data to test the accuracy of the estimated haplotype frequencies, assuming different levels of LoD_SNP_ and LoD_MOI._ These reflect realistic conditions of molecular detection in blood samples obtained from malaria patients. Since the simulation data represent the true situation among malaria patients, simulation results provide evidence that there is high confidence in the haplotype frequency estimation produced by any one of the statistical methods. However, there are differences between the statistical methods especially with increasing LoD_MOI_ and LoD_SNP_ that occur in real data. Even if the LoD_MOI_ are zero there is an impact on the observed MOI caused purely by chance when clones share the same MOI allele: the population MOI in misclassified (under-estimated) in around 5 % of patients. This directly effects the estimation of the haplotype frequency. That is the main limitations for the efficient use of haplotype frequency estimation in current, large scale, genetic epidemiology data among malaria patient.

The main difference between the statistical methods is how they deal with the number of infections, number of copies of haplotype (haplotype combination). The MHF (MalHaploFreq) cycles through all the combinations of haplotypes that can occur within that multiplicity of infection. The EM-algorithm implemented as R-EM (malaria.em) is based on the assumption that each patient has a fixed MOI. The Bayesian algorithm (Bayesian statistic) is based on model specification the selects a prior distribution of MOI based on one of four possible distributions (Uniform, Poisson, negative Binomial, and Geometric). The novel methods (EM and MCMC) used MOI estimates that were assumed to be provided for each patient and that clones within the MOI were acquired at random, so that the distribution of infections within patients is multinomial with the sample size of the MOI and frequencies of haplotype provided by their current estimates within the algorithm. The differences between the methods directly affect their results. The Hill climbing algorithm (MalHaploFreq) is accurate and valid when the LoD_MOI_ and LoD_SNP_ are zero, but increased LoD dramatically decreased the accuracy and the validity of the result (Fig. 5). The EM-algorithm implemented in R-EM (malaria.em) with the fixed MOI presents the opposite i.e. its accuracy and the validity increases when the LoD_SNP_ and LoD_MOI_ increases. The Bayesian (Bayesian statistic) with the prior distribution of MOI appears exhibits accurate and valid when the LoD_SNP_ and LoD_MOI_ are zero, but increasing the LoDs dramatically decreases the accuracy and the validity of the results. The novel EM-algorithm method obtains highly accurate and valid results irrespective of the LoD_SNP_ and LoD_MOI_ values. The MCMC method obtains results that are sensitive to LoD levels: its accuracy and validity both decrease as the LoD_SNP_ and LoD_MOI_ increase. Adding the constant *k* (1/MOI) to the algorithm preferentially weights the low-frequency haplotypes which should be reflected in slight overestimates of their frequency. Additional file 1: Figure S7 demonstrates the impact of several values of *k* i.e. 0, 0.01, 0.05, 0.1, 0.2, and 0.5 among the EM-algorithm. The correlation coefficient (*R*^*2*^) is slightly higher by the *k* = 1/MOI. Additional file 1: Figure S8 limits the X-axis scale to show the deviation of the estimated haplotype frequency among RAF <0.15 from population or sample haplotype frequency. The correlation coefficient (*R*^2^) is higher in the EM estimated haplotype frequency with correction *k* = 1/MOI compared to using EM without correction i.e. 6.24 % compared to 10.71 %.

The same basic approach is used in this EM-algorithm and that used in MalHaploFreq i.e. all possible combinations of haplotypes within a genotype are examined to obtain a likelihood of observing the dataset given current estimates of haplotype frequencies. Consequently, the results are very similar: correlation coefficient of the 1000 estimates obtained by the two methods is *R*^2^ = 0.98. However the EM-algorithm is much faster because the combinations are only generated once prior to the estimation steps, plus the 95 % CI are calculated algebraically in the EM-algorithm (Eq. 1) whereas MalHaploFreq uses a rather crude (and slow) hill-descending algorithm to define the CI as −2 LL units less than the maximum LL. The speed of the EM-algorithm is considerably faster than MHF (although MHF could be made considerably faster if it estimated only haplotype frequencies then calculated 95 % CI using Eq. 1, rather than its hill-descending algorithm). Speed becomes important because both methods suffer from one potential problem: they seek to identify the haplotype frequencies that provide best prediction of observed data using an interactive process that gradually increases the likelihood of observing the data. The problem is that this “hill climbing” process may converge onto a local “peak” of likelihood and miss a peak of higher likelihood located some distance away in parameter space. The consequence is that both methods need to be started from a large number of different parameter values to check that a single peak of likelihood is always identified and, if not, to ensure the analysis returns the haplotype frequency estimates obtained at the maximum peak. This potential problem was investigated by Hastings and colleagues [1, 4] who analysed datasets and reported this problem of multiple peaks existed in the analyses. The problem of multiple peaks is unpredictable so users are urged to analyze their dataset using a large number (1000 seems reasonable) of initial haplotype frequency estimates. This problem not exist in the EM-algorithm presented here, it can start from any set of initial starting frequencies, use random starting frequencies to check the algorithm converged on the same final estimates and runs quickly so the time penalty should be negligible.

The EM-algorithm examines each possible haplotype combination in an individual that could plausibly give rise to his/her observed genotype. This mean that once the haplotype estimates are obtained it would be possible to use them to obtain the probability that any given patient harbours a “drug resistant” haplotype and make clinical decisions on this basis. Whether data could be collected and analysed in a sufficiently timely manner for this to occur is debatable but consider it a point worth making. More plausibly, the presence of putative “resistant” haplotype can be inferred in individual patients and the probability of their presence used as a weighting in a logistic regression predicting the therapeutic outcome (cure/fail) of drug treatment. A positive impact of the putative-resistant haplotype on therapeutic outcome would be indicative that it truly does affect resistance levels.

Given the potential importance of MOI estimates, it is unfortunate that some surveys do not collect it [24–26]. The R-EM, Bayesian, EM and MCMC can calculate the haplotype frequency when MOI information on a patient was unknown (i.e. unmeasured or missing). Every one of the methods makes a prior assumption on the probability distribution on the number of infections per individual. The R-EM algorithm assumes MOI follows a Poisson distribution with mean = 2, the Bayesian algorithm can assume one of four possible distributions (Uniform, Poisson, negative Binomial, and Geometric). The EM and the MCMC algorithm described here, analyses datasets that were simulated assuming the frequency distributions that given by Jaki et al. [8]. The EM and the MCMC algorithm (Additional file 1: Figures S9, S10) obtain more accurate frequency estimates, and are slightly less affected by LoD of the SNPs and the MOI than compared to the related statistical approaches. Another option of the MCMC algorithm includes update to the MOI could be proposed when patient MOI is unknown and accepted/rejected during the updating stage. Four different algorithms runs with changing the MOI, present in the Additional file 1 MCMC algorithm 2, 3 and 4. Initial analyses suggested updating the MOI during MCMC made little or no difference to the resulting haplotype frequency estimates (Additional file 1: Figures S11–S14). A potential additional advantage is that the MOI distribution would be updated each iteration and hence the MCMC algorithm naturally provides an estimated distribution of MOI frequencies. However, the estimated MOI distribution did not reliable recover the population MOI and the analyses suggested more weight was given to MOI = 2 (60–80 % of the MOI distribution) in those algorithm which did update MOI (i.e. algorithms 2, 3 and 4; Additional file 1: Figures S15–S18). In retrospect this is not surprising: all the methods seek to provide the simplest explanation of the data and any heterozygous genotype can always be explained most parsimoniously by two haplotypes in the blood sample; the fact that some estimates of MOI are greater than 2 presumably reflects the situation a “2-haplotype” explanation is untenable due to the low frequency of the two plausible haplotypes. One consequence of this is that it appears impossible to analyse SNP data to obtain a MOI distribution using the MCMC Algorithms described here. This is unfortunate as MOI distributions are useful epidemiological indicators, high MOI values tending to reflect higher transmission intensities, and may be useful to asses, for example, the impact of malaria control measures on malaria transmission rate. It may be possible to recover more accurate MOI distribution by forcing a distribution onto the data, typically a Poisson or Negative Binomial. This was done by previous authors but was not attempted here as the intention was to avoid having to pre-specify a MOI distribution and simply let the MCMC algorithm find the best explanation for the data. It did this with considerable success, the haplotype frequency estimates showing excellent correlation with the population/sample values even in the absence of MOI information.

The study was conducted within a number of constraints imposed to ensure they appropriately address key questions in malaria research. The simulations were limited to two and three SNPs to simplify the comparison (results from haplotypes defined at three SNPs are presented in Additional file 1: Figures S19–S23, the same pattern irrespective of whether haplotypes are defined at two or three loci). This is consistent with previous analyses: MHF (MalHaploFreq) is limited to analysing up to three SNPs, the R-EM (malaria.em) can analyse more than three SNPs but required a considerable amount of computational time, and the Bayesian (Bayesian statistic) method, as implemented by Taylor et al. [6] is limited to handling up to seven SNPs. The novels methods described above did not have to limit the number of SNPs analysed but the examples were limited to three SNPs because the complexity of calculations rises exponentially with the number of SNPs and it is rarely necessary in practice to analyse more than three SNPs simultaneously [4] when investigating drug resistance haplotypes. However, calculating the frequencies of haplotypes that are defined at a large number of SNPs increases the computational time, the magnitude of this increase depending on the computer memory. It was considered important to recognize the technical limitations of genotyping so three values for levels of detection (LoD_SNP_ and LoD_MOI_) were investigated. The MOI distribution reflected the default frequency distributions given by Jaki et al. [8] and represents an area of relatively intense malaria transmission where MOI tends to be high and where the statistical problems of correctly estimating haplotype frequencies are most severe.

## Conclusion

In summary, the two novel methods proposed here have advantages over previous methods of inferring haplotype frequency. If MOI is known the EM algorithm appears the most natural way to analyse the data. It is explicitly set up to incorporate MOI data on individual patients (in contrast to EM-R and Bayesian) and is much faster than MHF. It also appear robust to chance misclassification of MOI and to genotyping detection limits (e.g. Figure 5) If MOI information is absent, the MCMC algorithm seems a more natural way of analysing data as it allows the algorithm to fit individual MOI to each patient rather than, as in R-EM and Bayesian, forcing a pre-determined distribution (Poisson or negative Binomial) onto the MOI; it appears that, at least in the simulations analysed here, that MOI is underestimated (Additional file 1: Figure S15) but the accuracy of haplotype estimates is maintained and is comparable to the other methods (Additional file 1: Figures S11, S12). In addition, isolating MCMC from its usual Bayesian context means that decisions on prior distributions of haplotype frequency and MOI distribution can be avoided as the MCMC converges on accurate estimates of haplotype frequencies irrespective of initial assumptions of haplotype and MOI frequencies. The R code used for these simulations and analyses are freely available on request to GKD.

## Additional file

10.1186/s12936-016-1473-5 Data that support and expand some of the interpretations and conclusions drawn in the main text, but whose inclusion would detract from the main argument.

## Abbreviations

EM: expectation–maximization
MCMC: Markov chain Monte Carlo
MOI: multiplicity of infection
RAF: resistance allele frequencies
LoD: limit of detection
MHF: MalHapFreq
R-EM: malaria.em
MH-MCMC: Metropolis–Hastings Markov chain Monte Carlo
ML: maximum likelihood
LE: linkage equilibrium
SNPs: single nucleotide polymorphisms
LL: log-likelihood
*I*_*F*_: similarity index
MSE: mean squared error
CI: confidence intervals
