# Supplementary material for: Markov chain Monte Carlo and expectation maximization approaches for estimation of haplotype frequencies for multiply infected human blood samples
Source: Malar J. 2016 Aug 25;15(1):430. doi: 10.1186/s12936-016-1473-5 (PMC4997664; doi:10.1186/s12936-016-1473-5)

Additional file 1:

Supplementary

This Additional file includes data that support and expand some of the interpretations and conclusions drawn in the main text, but whose inclusion would detract from the main argument.

The Expectation-Maximization (EM)-algorithm

The algorithm is an iterative method that consists of two steps i.e. an expectation step (*E*) and a maximization step (*M*) at each iterative [9, 10, 11, 12, 13]. The observed data consists of MOI and SNP genotypes (wildtype, resistant, mixed) at several genetic loci. The algorithm was developed to obtain the maximum likelihood estimates of the haplotypes and is applied to the observed genotype data with an initial assumption of the haplotype frequency. The distribution of haplotype in an individual is assumed to be multinomial with a sample size equal to the MOI value of that blood sample. Let n represent the number of blood samples (sample size), i is an index used to refer to an individual blood sample (i=1, …, n), j is an index used to refer to a unique haplotype combination within a blood sample i, $h_{(i,j)}$ is a set of haplotypes, s is the number of SNPs genotyped, q is the max MOI identified in the dataset, z is the number of potential haplotypes in the population (2^S), m is a vector of MOIs for each patient, $m = (m_1,...m_n)$. G is a vector of genotype group for each patient, $G = (g_1,...g_n)$. H is a vector of haplotype sets, $H = (H_1,...H^z)$ and θ is a vector of estimated haplotype frequencies, $\theta = (\theta_1,...\theta^z)$.

The Log-Likelihood (*LL*) is obtained by multiplying the probability of each individual. The probability of observing a particular genotype from an individual is the summation of all combination of haplotype frequencies for all haplotype infections that are consistent with the genotype. The complete data log-likelihood is give by:

$$LL_{(\theta)} = \Pr(G \mid H, h) \approx \prod_{y=1}^{z} \sum_{l=1}^{G} \log(\sum_{l=1}^{G} (\theta_y)^{H_y} \qquad \text{(S1)}$$

To reach the *LL* need to identify the sets of haplotypes that could give rise to the observed SNP genotype in a patient. This is achieved by systemically testing each combination of haplotypes within the MOI to identify the haplotype sets $h_{(i,j)}$ that can give rise to the observed patient genotypes.

The algorithm starts by specifying an initial set of estimated haplotype frequencies. This is held in a vector of haplotype frequency (θ) whose elements equal the total number of possible haplotypes (z). In the E-step (i.e. calculating the expectation of the complete data), the expectation is given by:

$$E_{(i,j)} = \prod_{y=1}^{z} (\theta_y + k_i)^{H_y} \tag{S2}$$

The factor k gives more weight to the lower frequency haplotypes and was included to try and improve the accuracy of their estimated frequencies (it may be a constant, such as 0.1 but by default we set its value as $1/m_\mathrm{i}$). Each possible haplotype combination within each patient is then multiplied by the current estimate of their haplotype frequencies to produce the variable $E_{(i,j)}$, assuming that the haplotype frequencies (θ) are true values.

The M-step maximizes the expectation of the complete data to update the estimated haplotype frequencies:

$$\theta = \sum_{i=1}^{n} \sum_{j} \frac{(G_i / n)}{\sum_{i \in G_i} E_i} E_{(i,j)} / m_i \tag{S3}$$

The G_i is the number of patients with genotype group i, $\Sigma_{i \in Gi}$ is summation of all individuals i that are in genotype group G_i. The M-step updates the estimated haplotype frequency: after a renewed estimation it returns to E-step and repeat the process until the estimated haplotype frequencies converge and remain constant. The iterations are tracked and convergence measured as:

$$diff = \frac{\Sigma_{\mathrm{z}} (\theta_{\mathrm{z}} - \widehat{\theta_{\mathrm{z}}})^2}{\Sigma\, \theta_{\mathrm{z}}{}^2} \tag{S4}$$

and iteration stopped when this metric falls below 1E-17.

The probabilities of possible haplotype of each individual in the sample are calculated as the posterior probabilities based on the estimated haplotype frequencies.

$$h_{(i)} = \frac{\prod_{y=1}^{z} \theta}{\sum_{(i)} \prod_{y=1}^{z} \theta} \tag{S5}$$

When MOI information on a patient was unmeasured or missing, the EM-algorithm proceeds as follows. To initialise the analyses each induvial is assigned an MOI according to the distribution frequency used by Jaki et al. [7] and all haplotype combinations within this MOI that give rise to the observed genotype are listed and processed. If no haplotype combinations can generate the observed genotype then another MOI is assigned, again using the MOI distribution of Jaki et al. [7]. The EM-algorithm then proceeds as described above. An alternative algorithm would be to list all the MOI and their possible haplotype combinations for a patient (and scaling equation S1 by the probability of that MOI in the population). Initial analyses using this approach incurred a very substantial speed penalty for a very small improvement in accuracy, hence out approach above i.e. that only a single MOI is investigated for each patient.

A detailed step-by-step guide describing how this algorithm is implemented and applied to malaria data sets is available on request from IMH.

The Markov Chain Monte Carlo (MCMC)-algorithm

The algorithm begins by assigning a sequence of haplotypes from a multinomial distribution (initial guess) that can give rise to the observed SNP genotype in each patient. These haplotypes are held in a matrix D whose size depends on the sample size (n, rows), and the maximum number of observed MOI (q, columns). The elements of this matrix are the haplotypes that are consistent with the observed patient genotype. For example if patient i is currently assumed to hold haplotypes 01, 10, 11 and 11 and maximum MOI is 8 then row i of matrix D would be {01,10,11,11,-,-,-,-} where '-' indicates no haplotype is required. The elements of matrix D can then be used to obtain the current estimates of haplotype frequencies by calculating haplotype proportions which are, stored in vector θ. The next step is to choose an individual at random from among those individuals with ambiguous genotypes (i.e. individuals where more than one sequence of haplotypes can give raise to the observed genotype). An update is proposed by simulating a new sequence of haplotypes consistent with observed genotype and MOI. Since this is a sequence it takes account of the fact that there can be several sequences all giving rise to the same haplotype combinations (do not need to include multinomial coefficients in the calculations). An update may be proposed (and accepted because Ψ=1; see below) even if both the original and update are different sequences within the same combination (e.g. {01, 10, 11, 11,-,-,-,-} may be replaced {10, 01, 11, 11,-,-,-,-}. The decision whether or not to accept the update depends on the following metric:

$$\Psi = \frac{\prod_{i=1}^{m_u} (\theta_{(h_i u)} + k)}{\prod_{i=1}^{m_o} (\theta_{(h_i p)} + k)} \qquad \text{(S6)}$$

Where m_u is the MOI of the proposed update sequence; m_o is the original sequence MOI, $h_i u$ is haplotype of the updated parameter; $h_i p$ is haplotype of the previous parameter. If $\Psi \geq 1$ then the update is always accepted, else Ψ is the probability of accepting the update. The addition of a constant k (1/MOI) gives more weight to low-frequency haplotypes and is discussed

more fully in the main text. At the end of each iteration the current estimate of haplotype frequencies in θ is updated. This process of proposing and accepting/rejecting updates until the estimated haplotype frequencies converged stationary distribution. Iterations defined as being completed when every heterozygous individual has been selected and tested for an update. Patients are selected at random (i.e. there is no set sequence) but every patient can only be selected one time in each iteration. The algorithm makes at least 100 iteration and the trace and autocorrelation output as graphs (supplementary Figures S24 and S25). The algorithm tested with run 100 iteration simulates multiple chains with different starting values and reached the same results. The trace plots the iteration number against the sampled values (theta) for each variable (haplotype) in the chain, with a separate plot per variable (haplotype). It can show when the chain gets stuck in certain areas of the parameter space, which indicates bad mixing. The autocorrelation is the correlation between the theta values in the current iteration with their values in the previous iteration. This enable the user to check that convergence to a stationary distribution has occurred, to identify the burn in period, and discount frequency estimates made during this burn-in period. This can be done by visual inspection of the graphs.

Suggested algorithm when MOI information on a patient is unknown

When MOI information on a patient is unknown (i.e. was not unmeasured or missing) the MCMC algorithm proceeds as follows. One difference to the analysis above is that when MOI can be updated in each patient (Algorithms 2 to 4) then homozygous individuals also need to be incorporated and updates proposed in each iteration: the haplotypes they contain will be the same, but their MOI may differ:

Algorithm 1: To initialise the analyses each individual is assigned an MOI according to

the distribution frequency given by Jaki et al. [7] and a combination of haplotypes is selected within this MOI that generates the observed genotype. If no combination within that MOI can generate the observed genotype, then the process is repeated until a MOI/haplotype combination is obtained that is consistent with the observed genotype. The patients' MOI are never changed during the MCMC updating and the algorithm proceeds as described above i.e. by assigning a sequence of haplotypes from a multinomial distribution that can give rise to the observed SNP genotype in each patient and accepted/rejected the proposed changes using Ψ as described above in Equation S6.

Algorithm 2: An update to the MOI could be proposed where patient MOI is unknown and accepted/rejected during the updating stage. The algorithm run with changing the MOI option when it suggests a new sequence of haplotypes consistent with observed genotype and MOI, the algorithm draws a new MOI according to the Jaki et al. [7] distribution; of note, the draw are always made from the MOI distribution of Jaki et al. throughout the iterations. All the other stages of the algorithm stay the same as described before and an update is accepted/deleted as described by Equation S6 above. Initial analyses suggested updating the MOI during MCMC made no difference to the results (Supplementary Figure S11). This would have the advantage that patients' MOI would be updated each iteration and hence the MCMC algorithm naturally provide distributions of both haplotype and MOI frequencies (Supplementary Figure S15); time precluded a fully investigation of this approach. The datasets are simulated assuming the MOI distribution of Jaki et al, and the MOI distribution used in the MCMC is also that of Jaki. This algorithm therefore analyses a situation where the MOI distribution in the population is known *a priori*. It therefore serves as a baseline for the two algorithms below, which update the MOI distribution each iteration and will drift away from the initial MOI distribution.

Algorithm 3: The MOI distribution is initially that of Jaki et al, but this distribution is updated at the end of each iteration to reflect the MOI distribution in the current iteration; the process is run until both the haplotype estimates and the MOI distribution have become stable. Each patient has an update proposed as follows. An updated MOI is suggested (which may be the same as the current MOI) according to the current MOI distribution in that iteration, and a haplotype combination within that updated MOI is identified that generates the required observed genotype of that patient. If no such combination is possible within the proposed, updated MOI then a new MOI is proposed and the process repeated until an updated has been identified that does reflect the patient genotype. The decision as to whether to accept the update is then:

$$\Psi = \frac{p(m_u)\prod_{i=1}^{m_u}(\theta_{(h_i u)} + k)}{p(m_o)\prod_{i=1}^{m_o}(\theta_{(h_i p)} + k)} \quad \text{(S7)}$$

Where m_u is the MOI of the update and m_o is the original MOI; $p(m_u)$ and $p(m_o)$ are their frequencies in the current MOI distribution; $h_i u$ is haplotype of the updated parameter; $h_i p$ is haplotype of the previous parameter. The initial MOI distribution is that of Jaki et al but it is updated each iteration so, in principle, any initial MOI distribution could be used.

Algorithm 4: The problem with the algorithm above is that it will favour the simplest MOI/genotypes consist with the observed data; hence they are likely to under-estimate the true MOI. For example, if a patient has only a single non-mixed genotype, for example [0,1] then the predicted MOI is likely to be 1 with a single haplotype [0,1] which would arise with frequency $\theta_{[0,1]}$; a values of MOI=4 is consistent with the data, but the probability of generating that genotype is $(\theta_{[0,1]})^4$ which is obviously far less likely to be accepted by the algorithms. The same applies to more complex, heterozygous genotypes so attempted to

offset this effect, at least partially, by allowing for the fact that different sequences can give rise to the same combinations e.g. [0,1]+[1,0]+[0,1] is the same combination as [0,1]+[0,1]+[1,0]. It is done using the multinomial coefficient:

$$\left(\frac{m_u}{k_1, k_2, \dots}\right) = \frac{m_u!}{k_1! k_2! k_3!} \quad \text{(S8)}$$

k_1, k_2 etc are the number of haplotypes of type 1,2 within the proposed genotype. So that:

$$\Psi = \frac{p(m_u)\left(\frac{m_u}{k_1, k_2, \dots}\right)\prod_{i=1}^{m_u}(\theta_{(h_i)} + k)}{p(m_o)\left(\frac{m_u}{k_1, k_2, \dots}\right)\prod_{i=1}^{m_o}(\theta_{(h_i)} + k)} \quad \text{(S9)}$$

Figure S1: The correlation (R^2) between population/sample and estimated haplotype frequency across statistical methods among LoDSNP=0.10, LoDMOI=0.05, the line through [0,1] represents the situation where both estimates are identical.

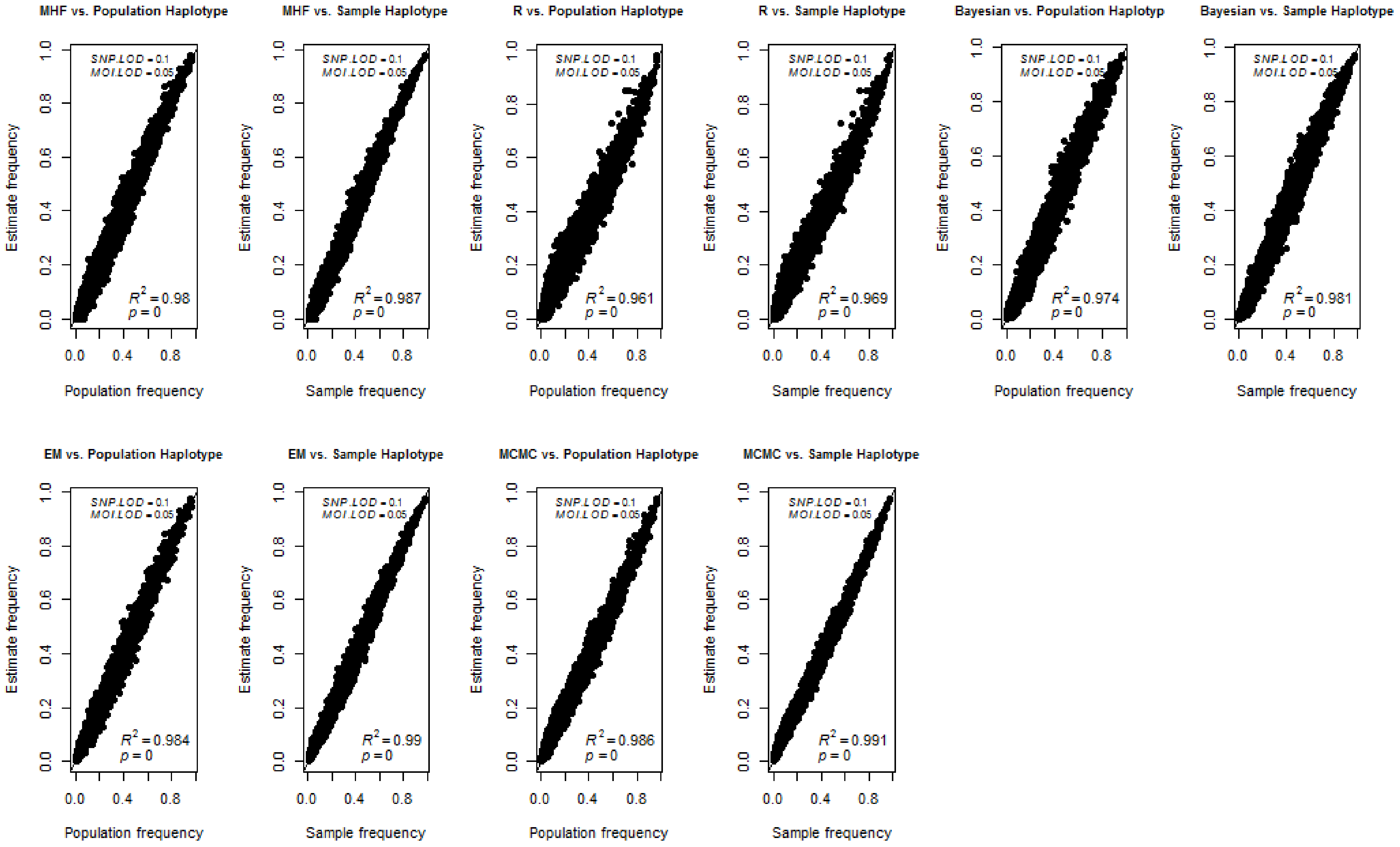

Figure S2: The correlation (R^2) between population/sample and estimated haplotype frequency across statistical methods among LoDSNP=0.20, LoDMOI=0.10, the line through [0,1] represents the situation where both estimates are identical.

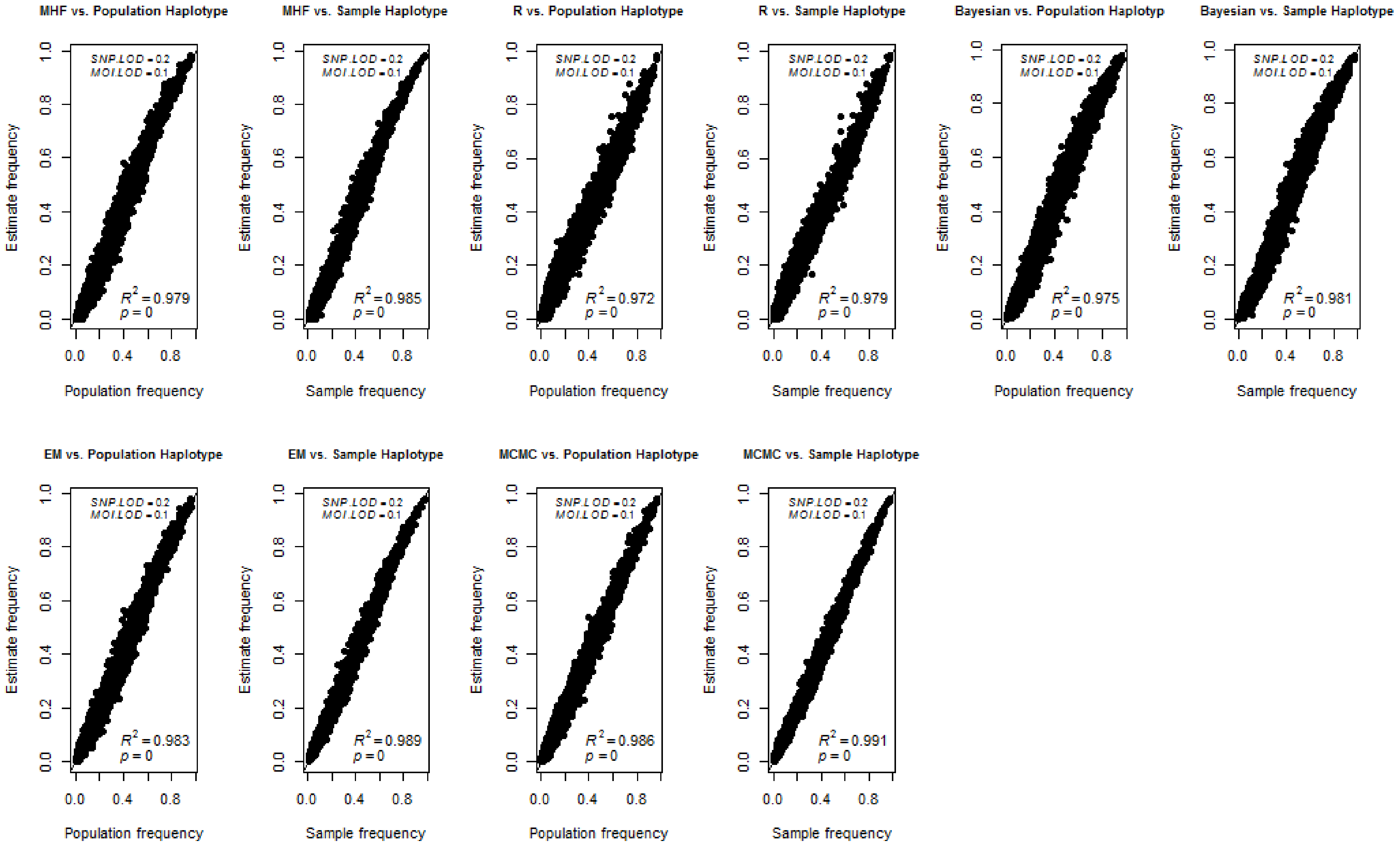

Figure S3: The correlation (R^2) between population/sample and estimated haplotype frequency across statistical methods among LoDSNP=0.30, LoDMOI=0.15, the line through [0,1] represents the situation where both estimates are identical.

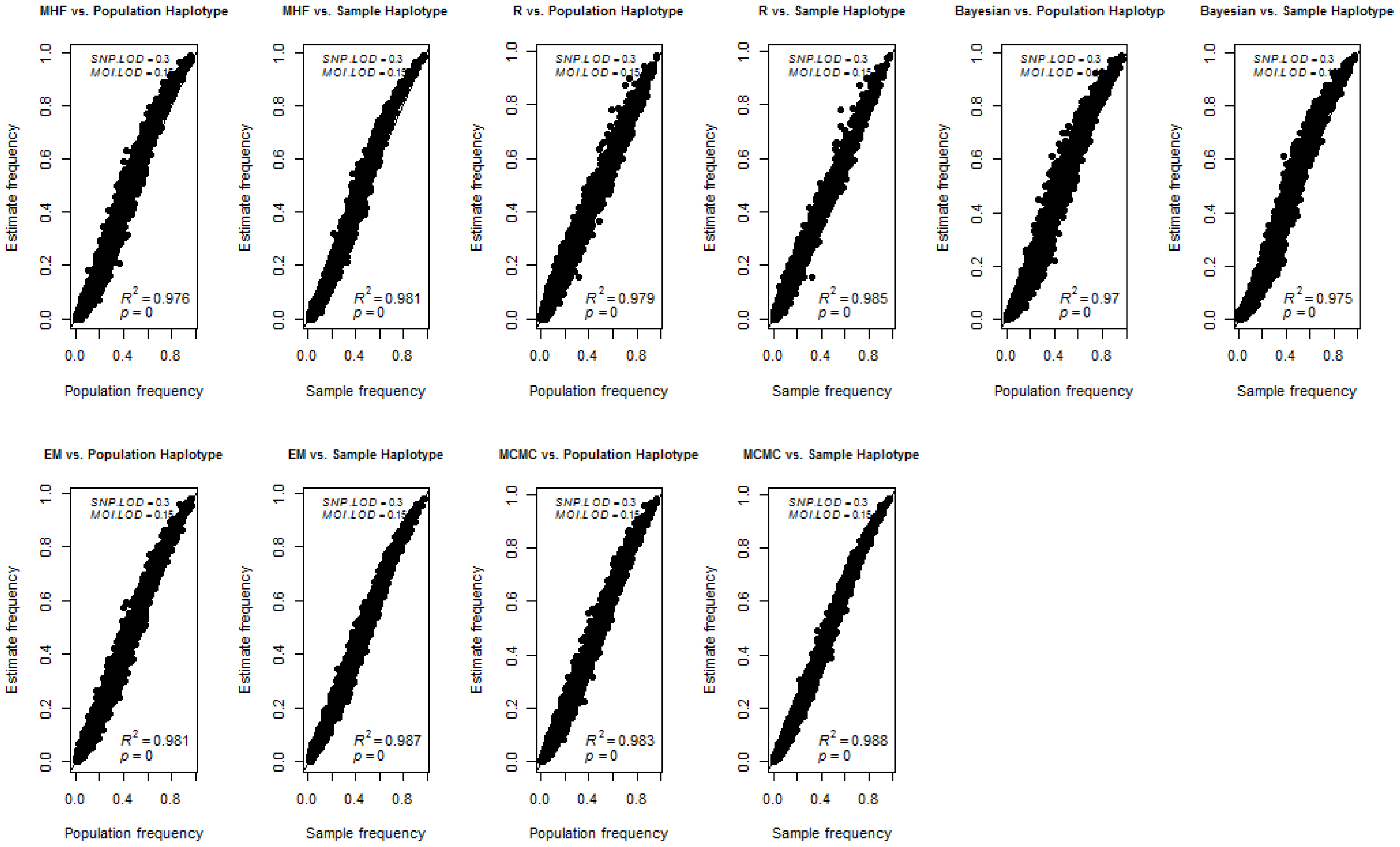

Figure S4: The change coefficient (*C*) between population/sample and estimated haplotype frequency across statistical methods among LoD_{SNP}=0.10, LoD_{MOI}=0.05.

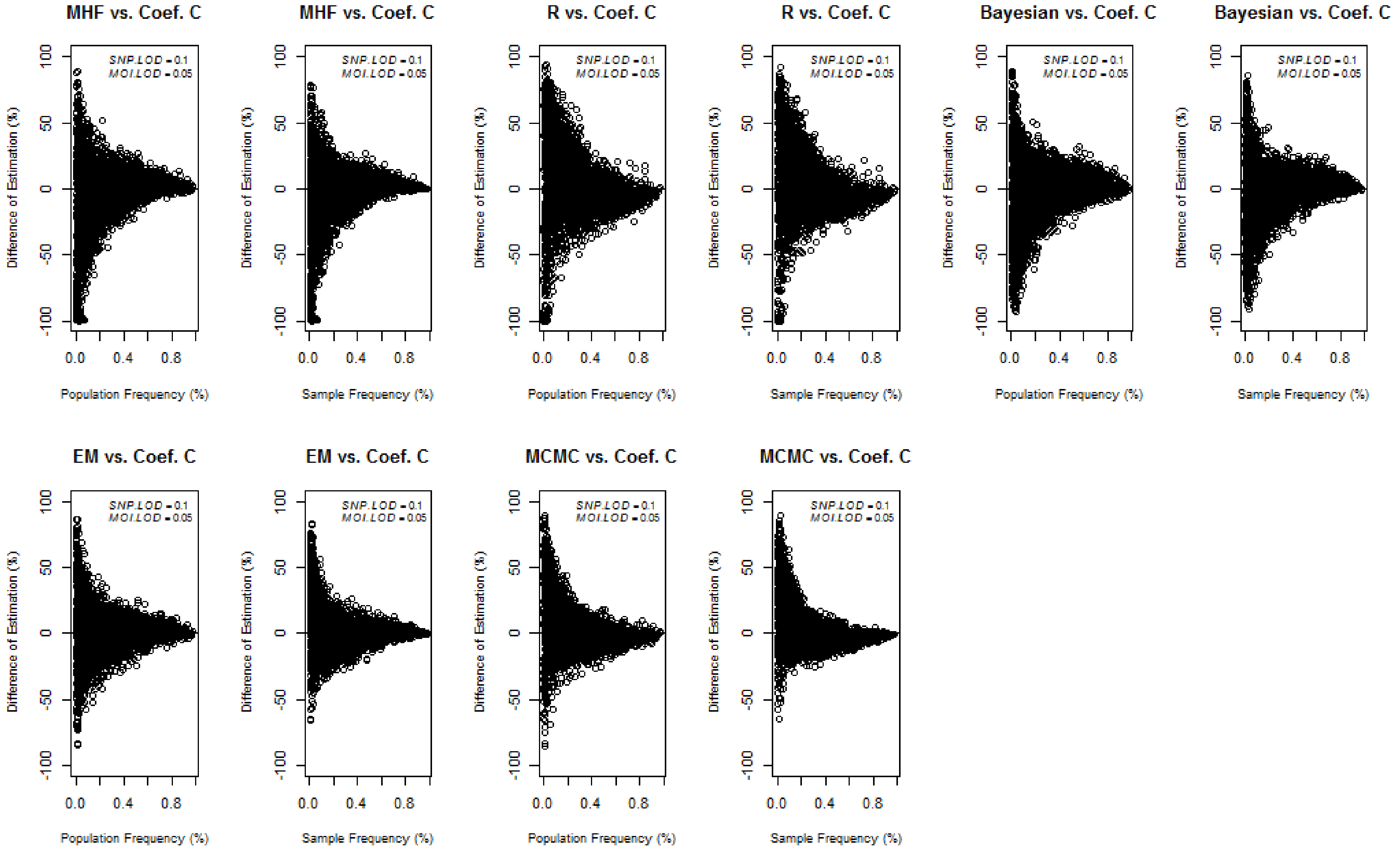

Figure S5: The change coefficient (*C*) between population/sample and estimated haplotype frequency across statistical methods among LoD_{SNP}=0.20, LOD_{MOI}=0.10.

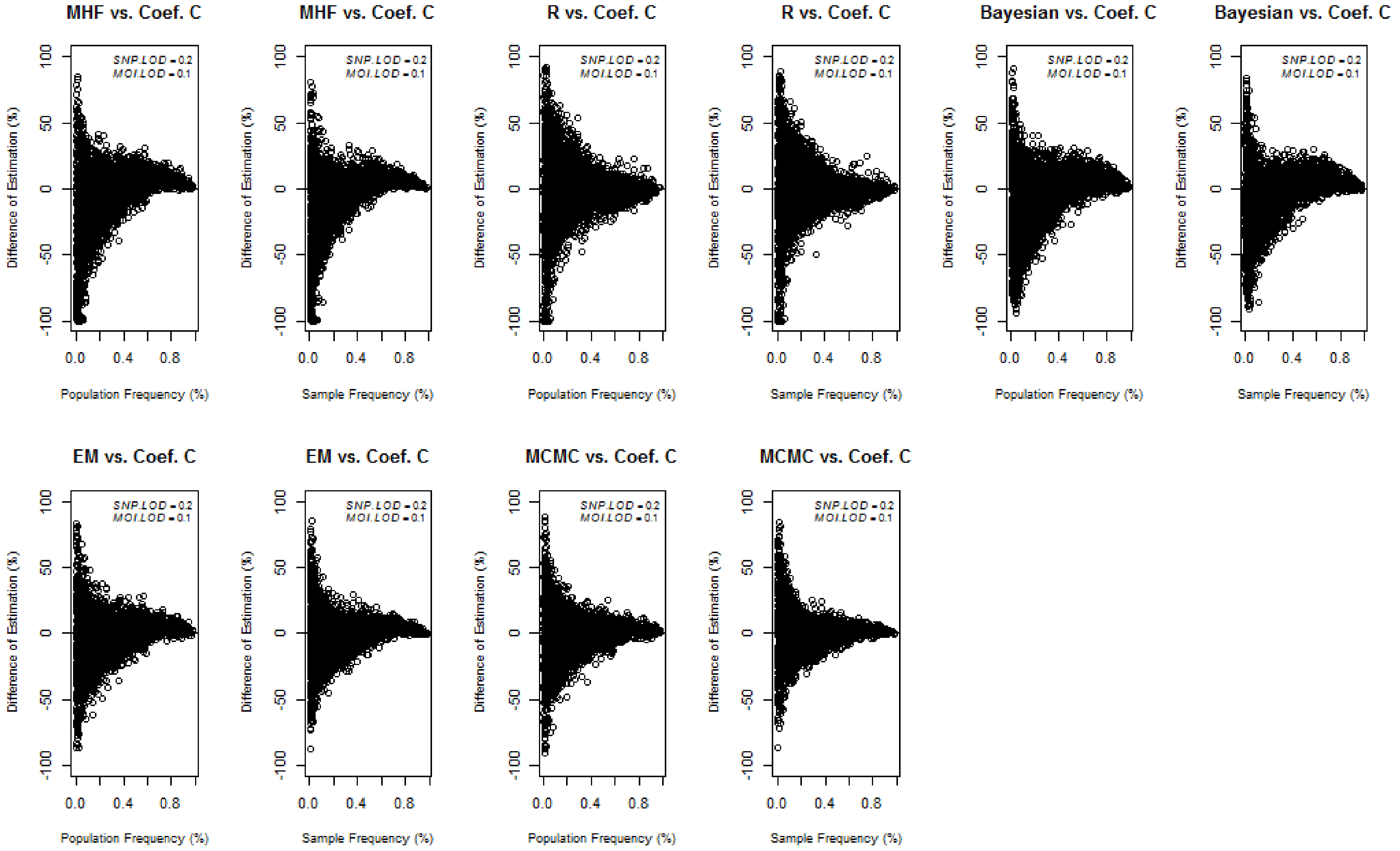

Figure S6: The change coefficient (*C*) between population/sample and estimated haplotype frequency across statistical methods among LoD_{SNP}=0.30, LoD_{MOI}=0.15.

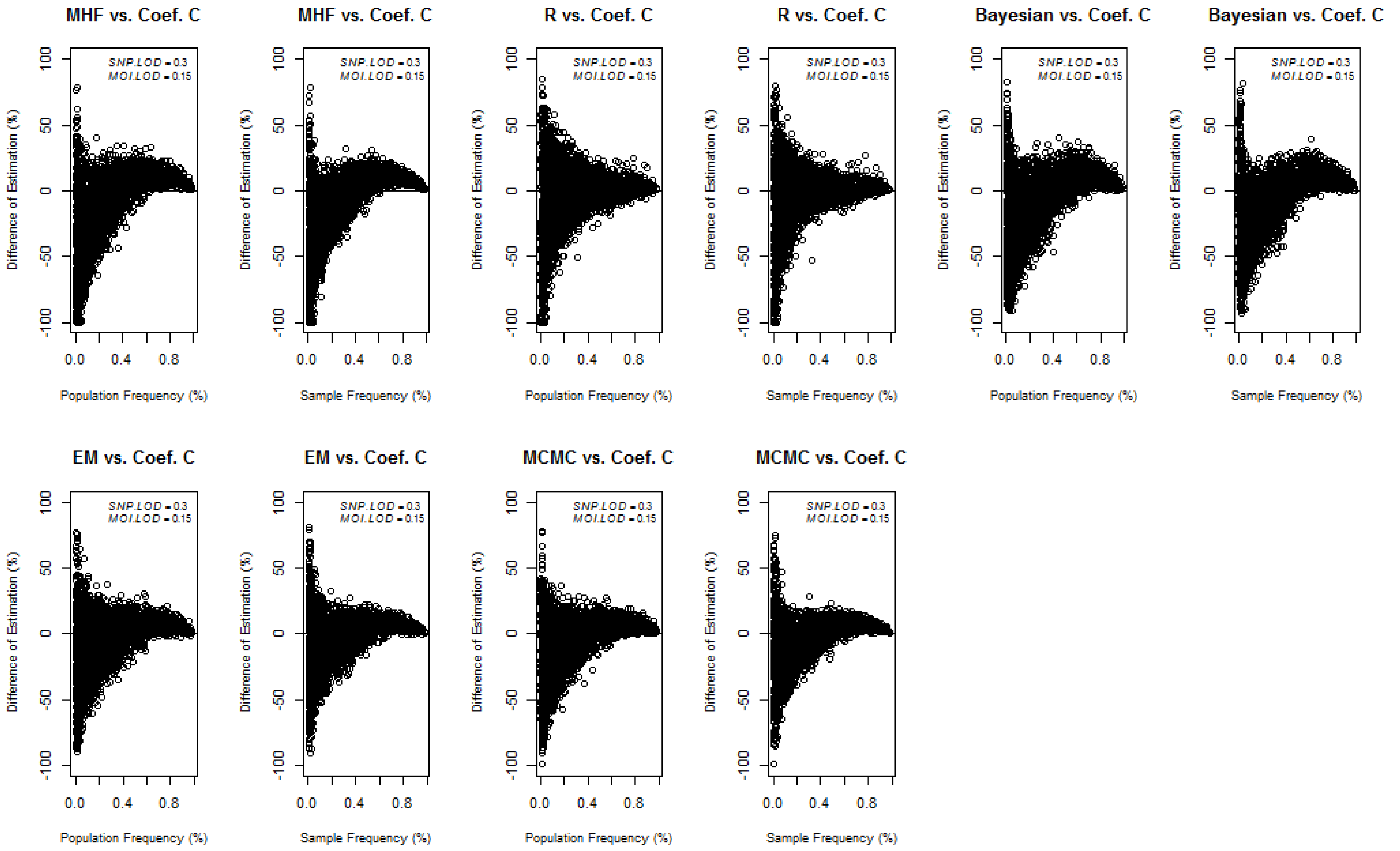

Figure S7: The correlation (R^2) between population/sample and the EM algorithm estimated haplotype frequency across different constant k (0, 0.01, 0.05, 0.1, 0.2, and 0.5) among LoDSNP=0.00, LoDMOI=0.00, the line through [0,1] represents the situation where both estimates are identical.

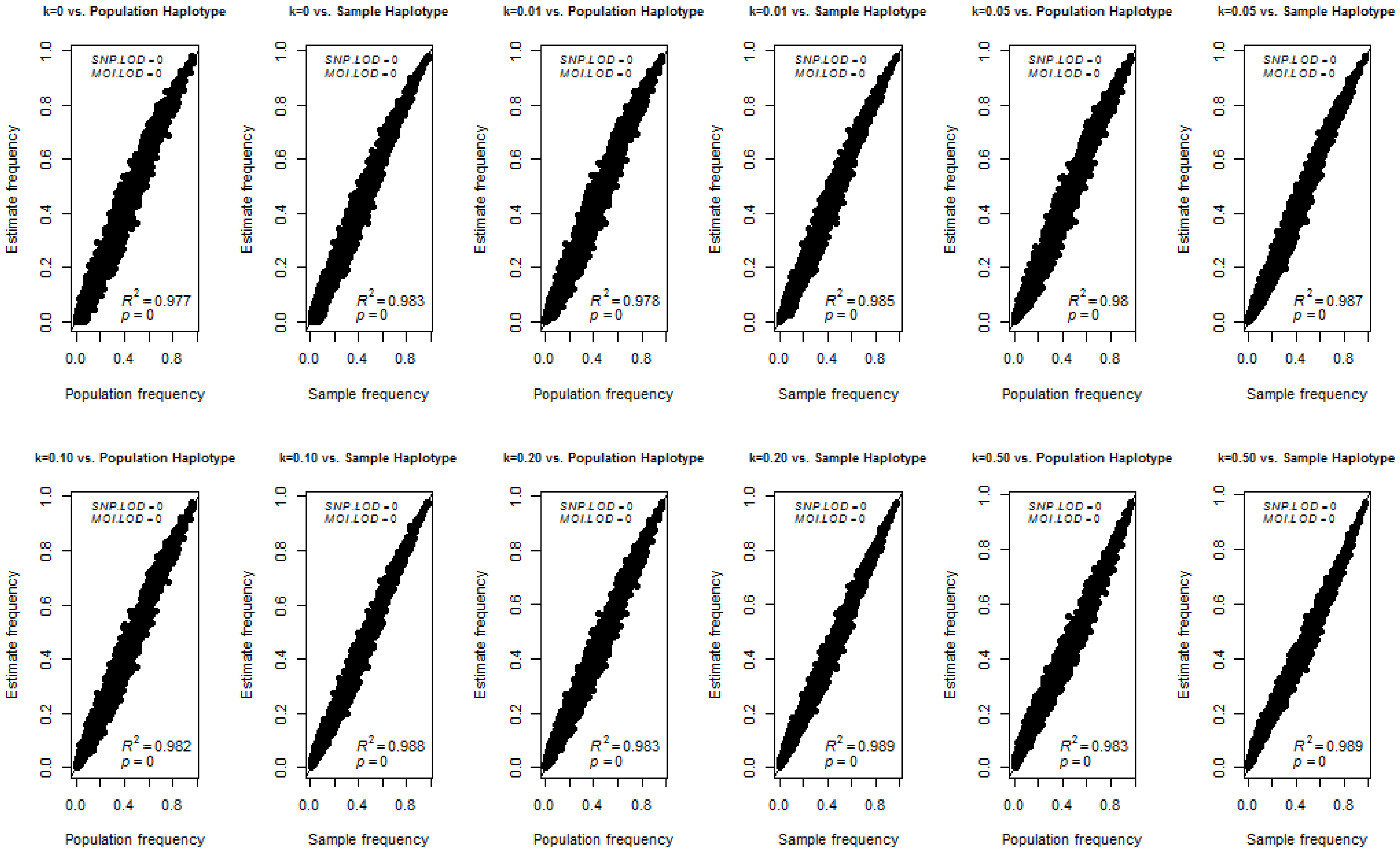

Figure S8: The correlation (R^2) between population/sample (RAF <0.15) and the EM algorithm estimated haplotype frequency across different constant k (0, 0.01, 0.05, 0.1, 0.2, and 0.5) among LoD_{SNP}=0.00, LoD_{MOI}=0.00, the line through [0,1] represents the situation where both estimates are identical.

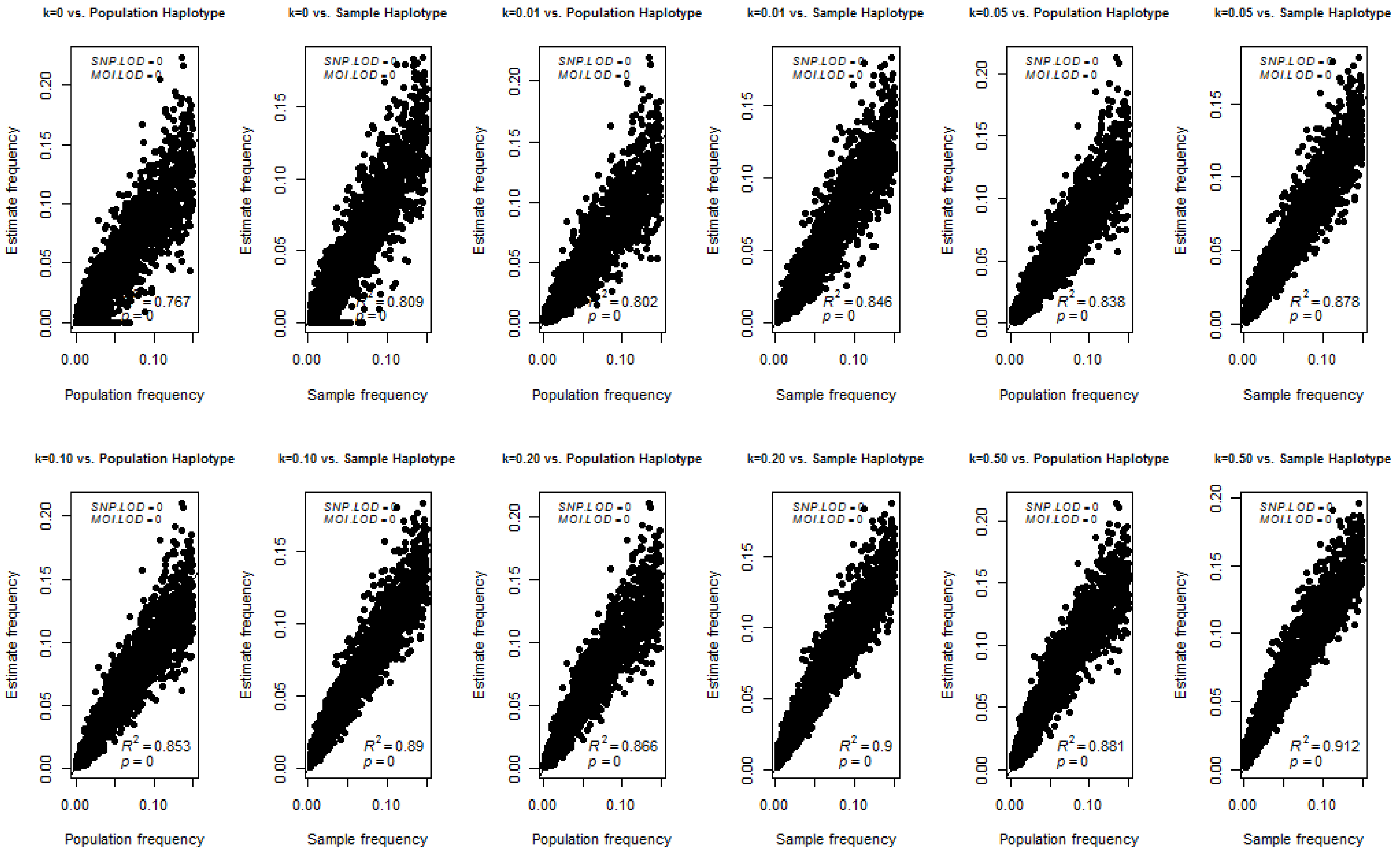

Figure S9: The correlation (R^2) between population/sample and estimated haplotype frequency, the line through [0,1] represents the situation where both estimates are identical among 2 SNPs, LoD_{SNP}=0, LoD_{MOI}=0 and unknown MOI (no results can be obtained from MHF because that method cannot deal with unknown MOI).

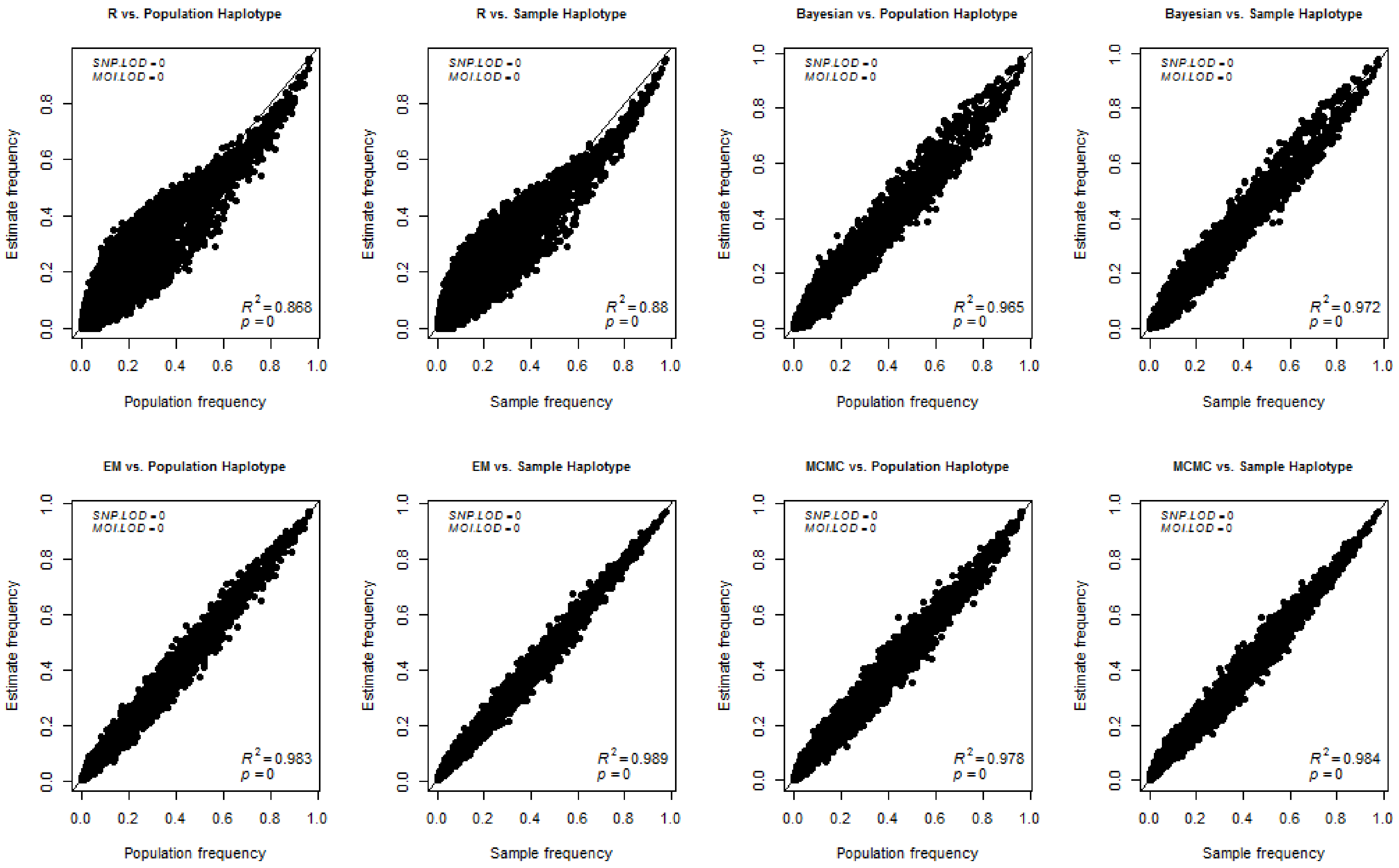

Figure S10: The correlation (R^2) between population/sample and estimated haplotype frequency, the line through [0,1] represents the situation where both estimates are identical among 2 SNPs, LoD_{SNP}=0.30, LoD_{MOI}=0.15 and unknown MOI (no results can be obtained from MHF because that method cannot deal with unknown MOI).

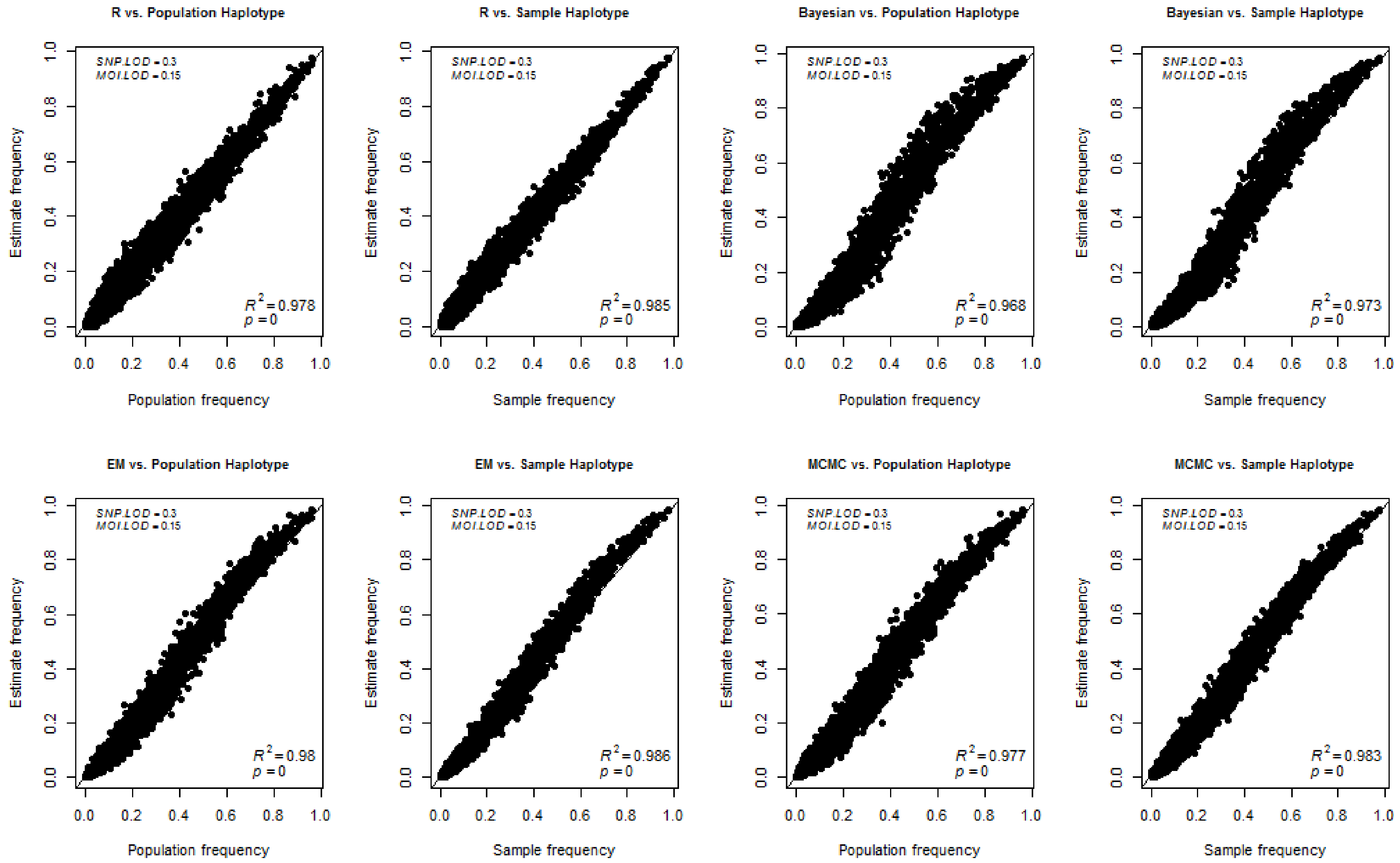

<u>Figure S11:</u> The correlation (R^2) between estimated and population/sample haplotype frequencies. The patients' MOI were assumed to be unknown and the results were obtained using MCMC Algorithm 2.

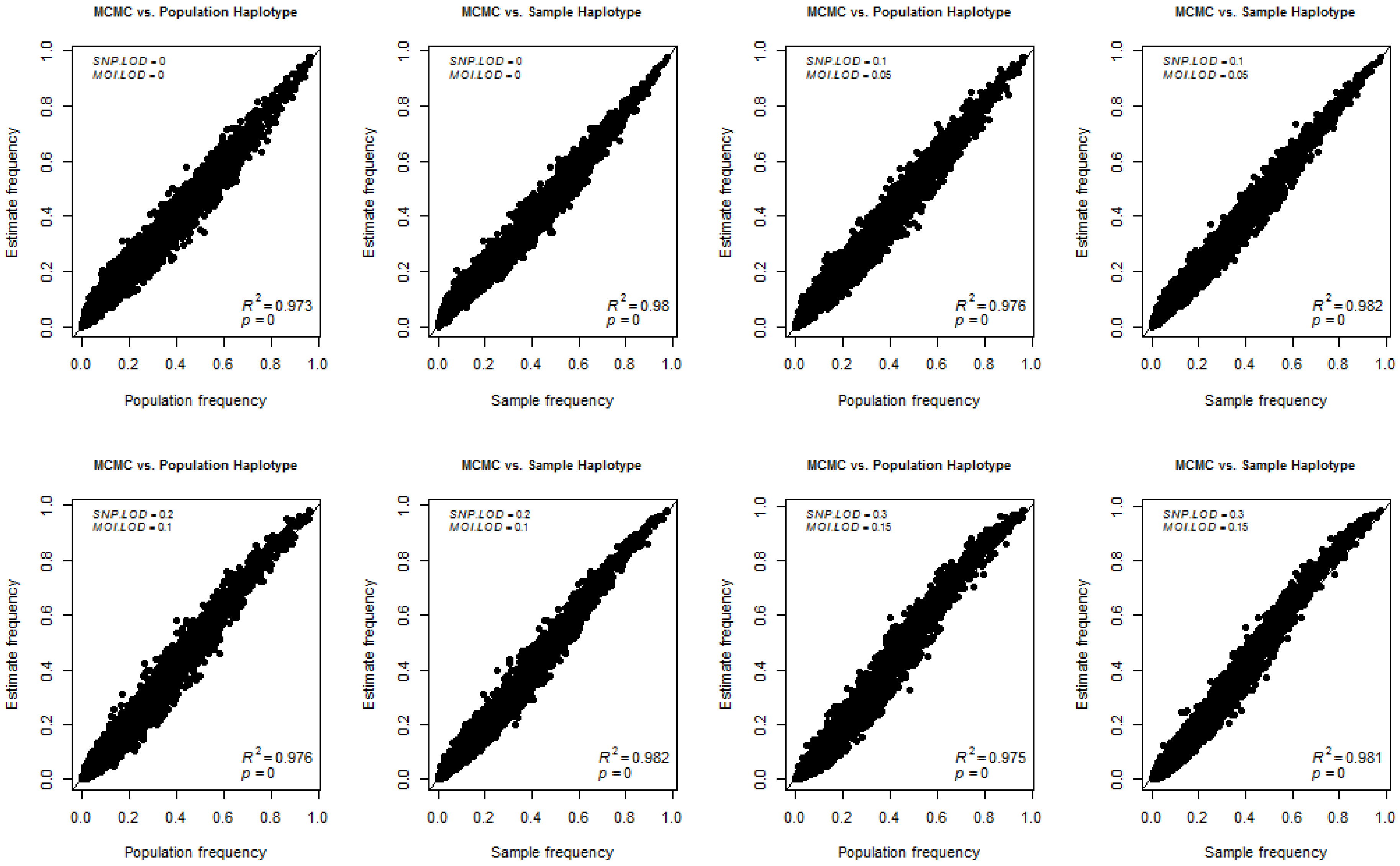

Figure S12: The correlation (R^2) between estimated and population/sample haplotype frequencies. The patients' MOI were assumed to be unknown and the results were obtained using MCMC Algorithm 3 using an initial distribution of Jaki et al.

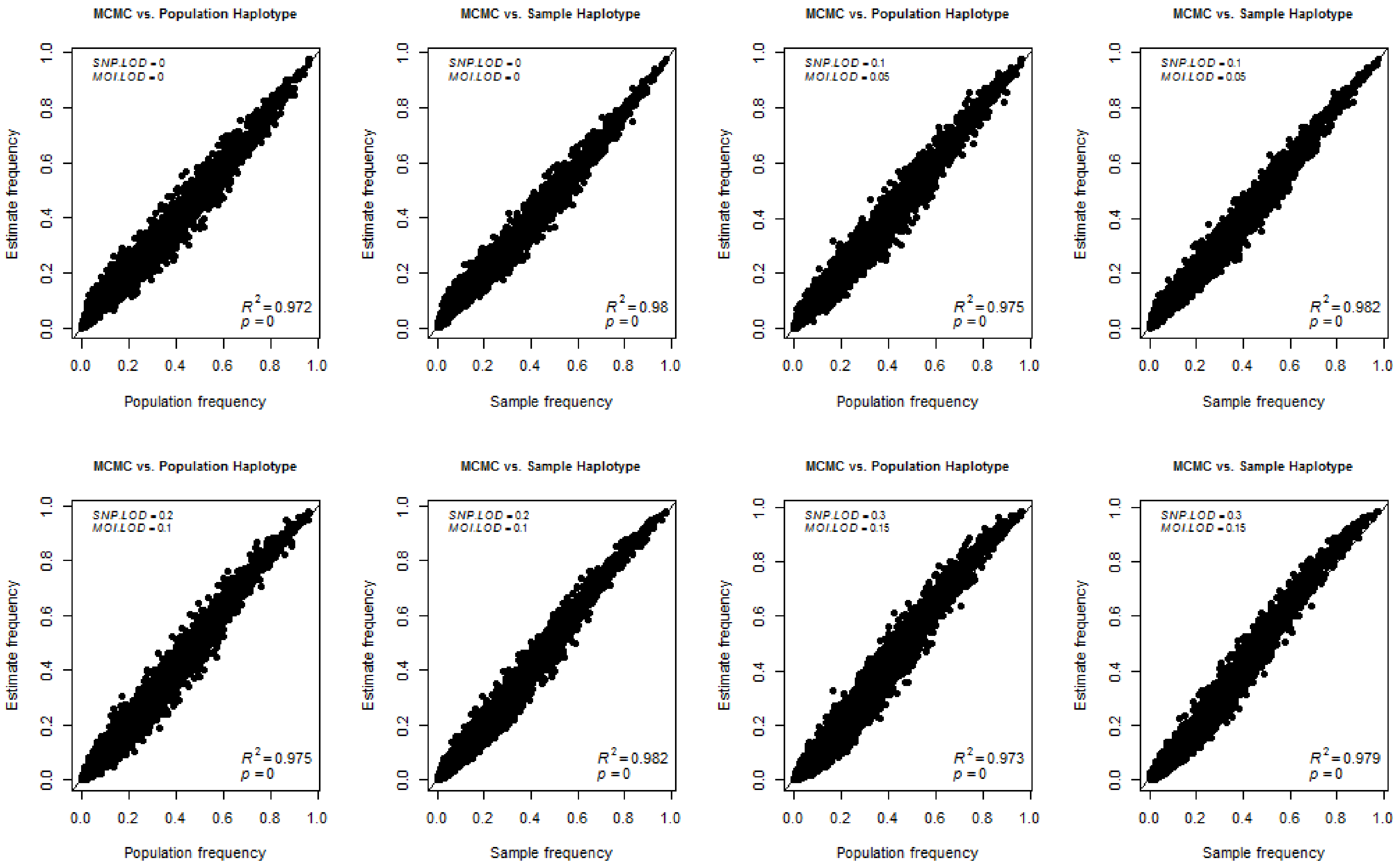

Figure S13: The correlation (R^2) between estimated and population/sample haplotype frequencies. The patients' MOI were assumed to be unknown and the results were obtained using MCMC Algorithm 3 using an initial MOI distribution that was uniform across MOI.

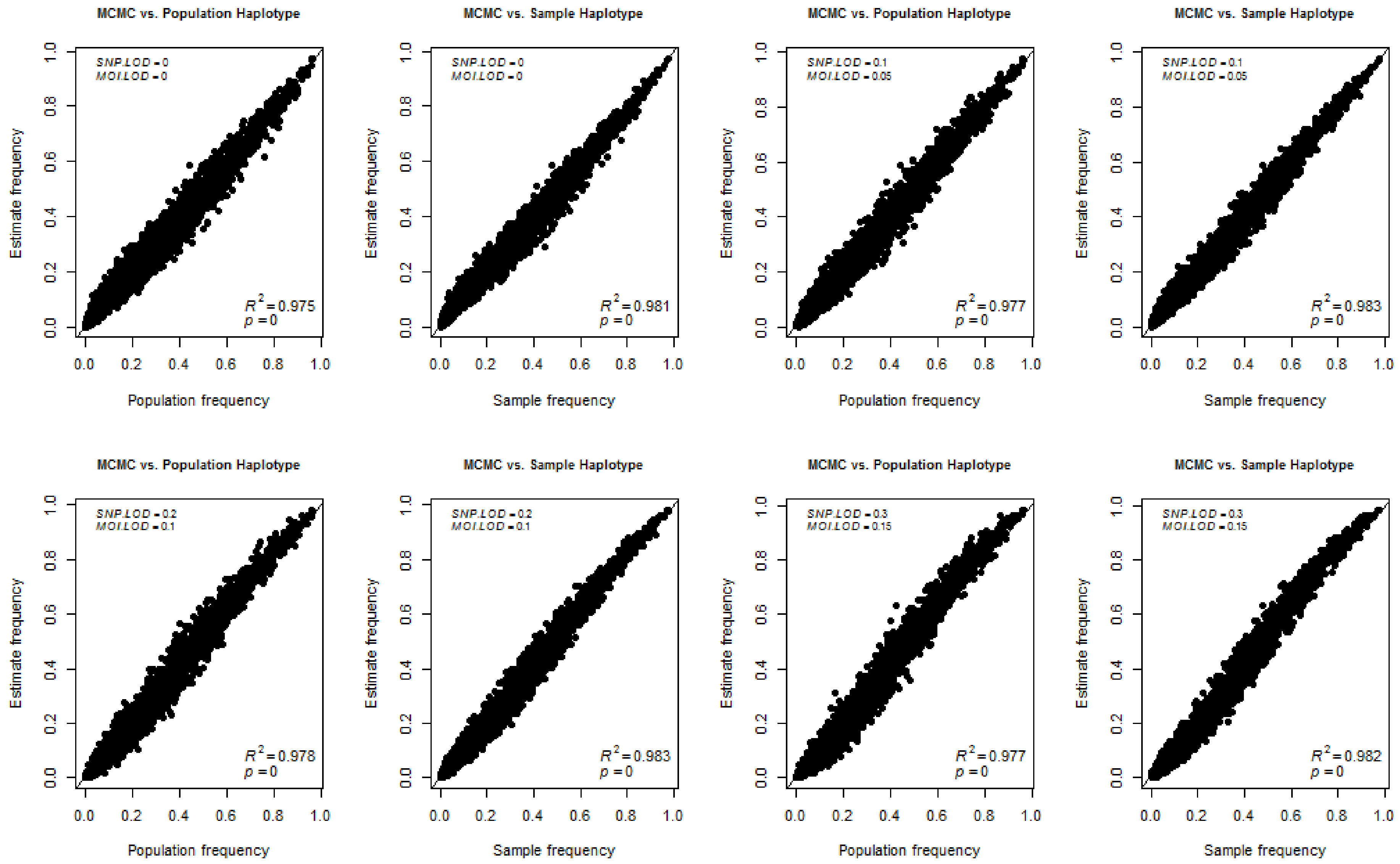

Figure S14: The correlation (R^2) between estimated and population/sample haplotype frequencies. The patients' MOI were assumed to be unknown and the results were obtained using MCMC Algorithm 4 using an initial distribution of Jaki et al.

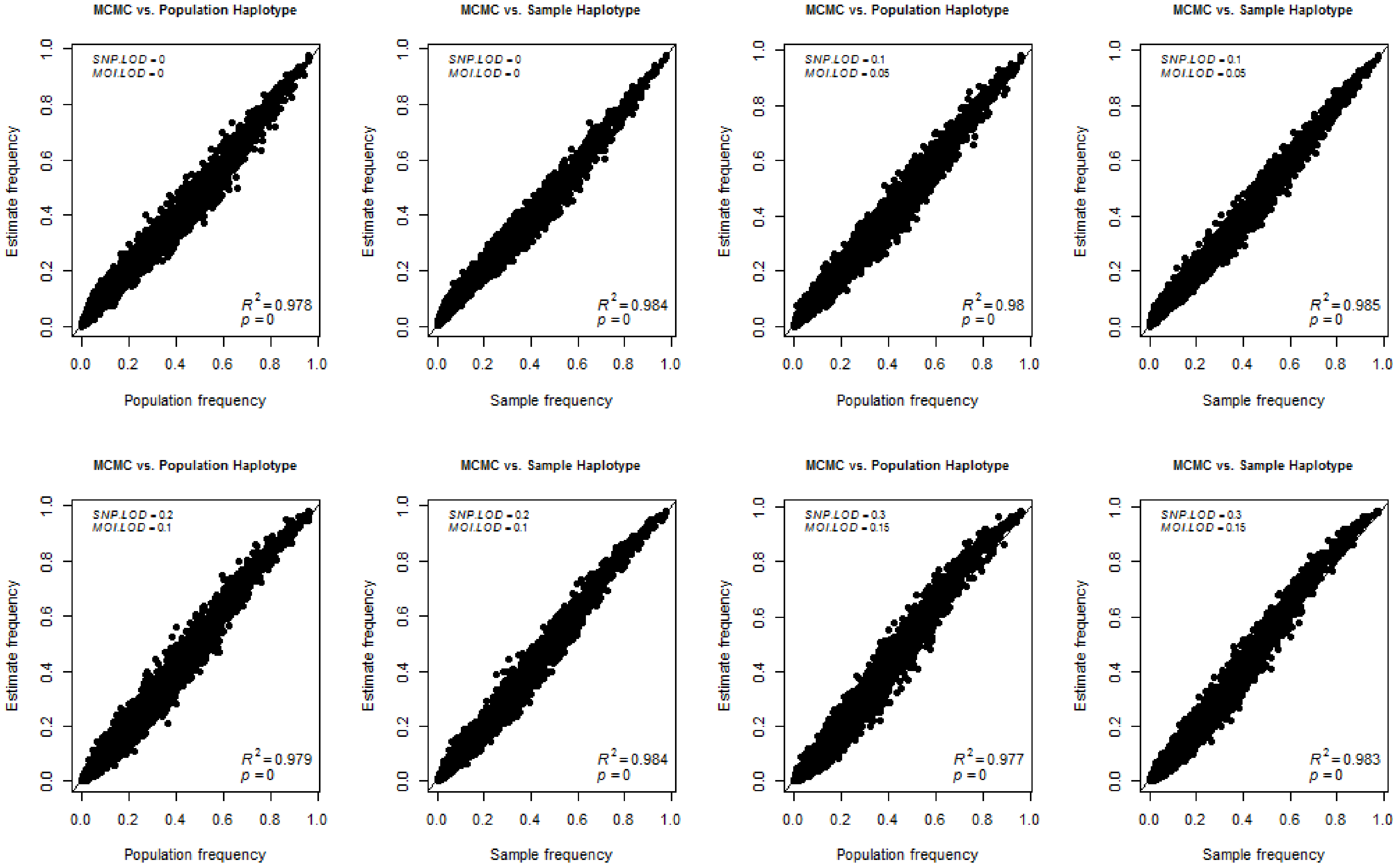

Figure S15: The MOI frequency distributions in the whole patient population ("Population MOI"), the patient population in the sample under analysis ("Sample MOI") and the distribution estimated by the MCMC algorithm allowing for different levels of genetic detection i.e. LoD_{SNP}=0%/10%/20%/30% and LoD_{MOI}=0%/5%/10%/15%. This came from algorithm 2 (i.e. start with Jaki et al distribution) mean after 100 iterations.

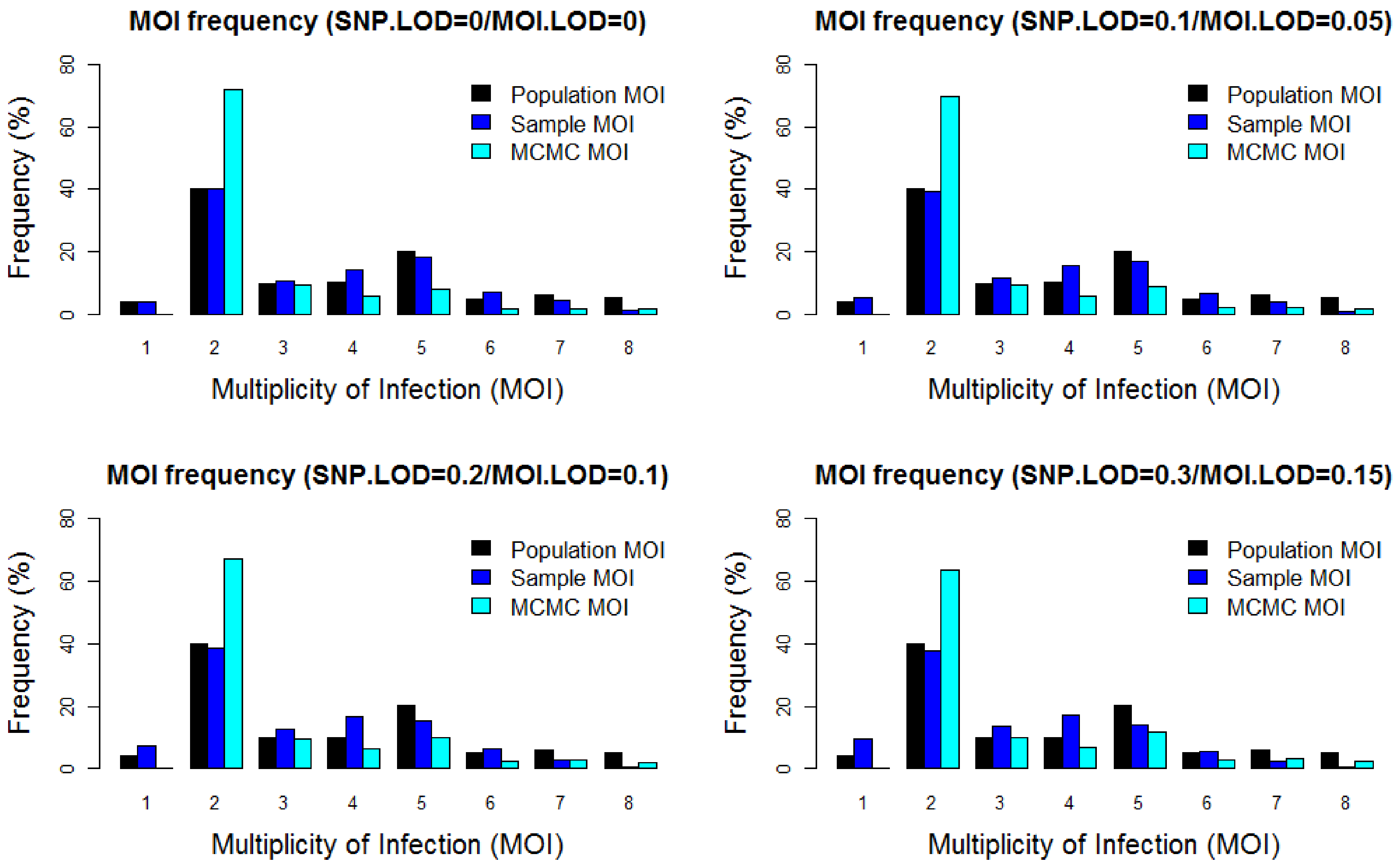

Figure S16: The MOI frequency distributions in the whole patient population ("Population MOI"), the patient population in the sample under analysis ("Sample MOI") and the distribution estimated by the MCMC algorithm (with change MOI) allowing for different levels of genetic detection i.e. LoD_{SNP}=0%/10%/20%/30% and LoD_{MOI}=0%/5%/10%/15%. This came from algorithm 3 (i.e. always use a Jaki et al distribution) mean after 100 iterations.

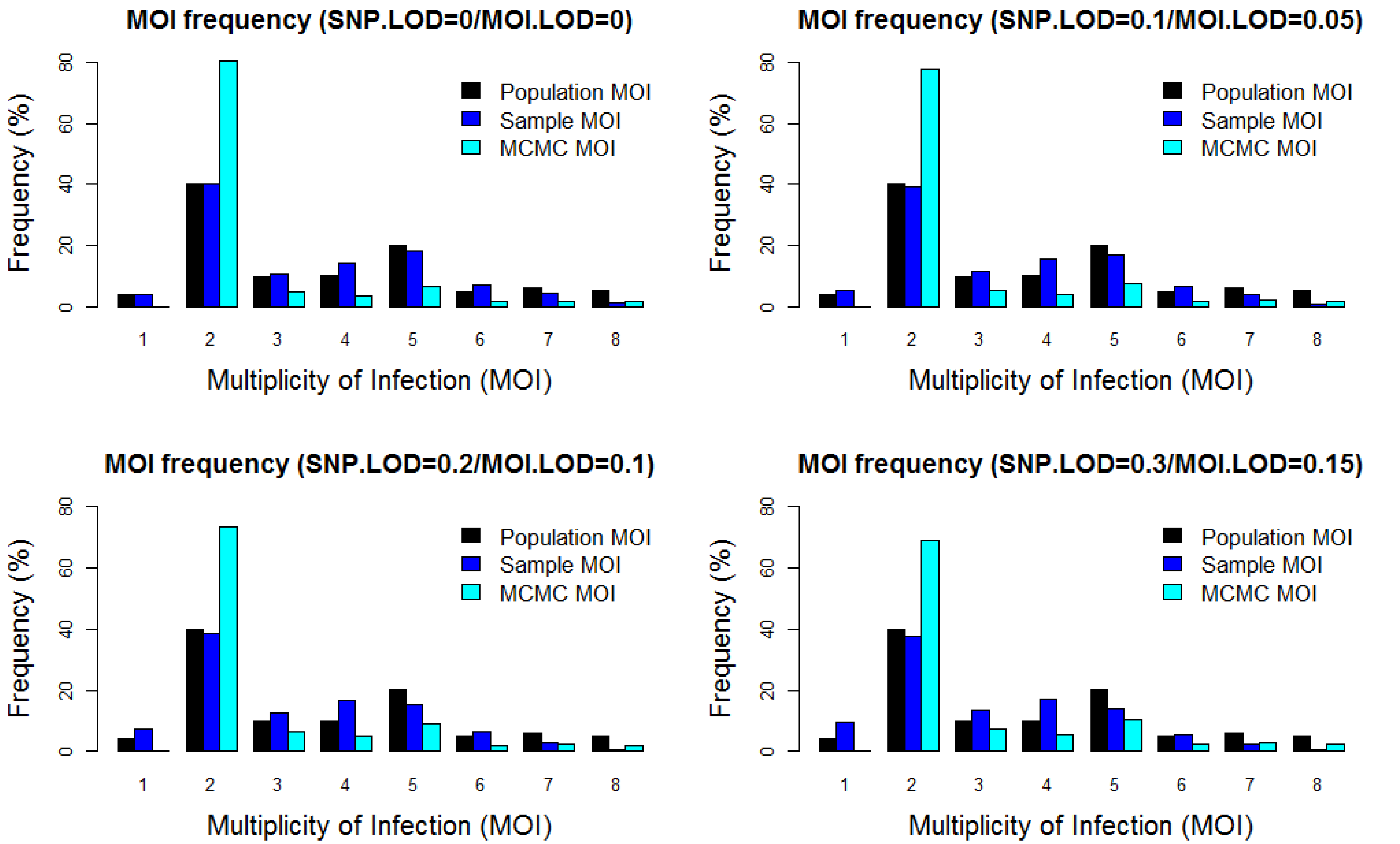

Figure S17: The MOI frequency distributions in the whole patient population (“Population MOI”), the patient population in the sample under analysis (“Sample MOI”) and the distribution estimated by the MCMC algorithm (with change MOI) allowing for different levels of genetic detection i.e. LoD_{SNP}=0%/10%/20%/30% and LoD_{MOI}=0%/5%/10%/15%. This came from algorithm 3 (i.e. always use a uniform distribution) mean after 100 iterations.

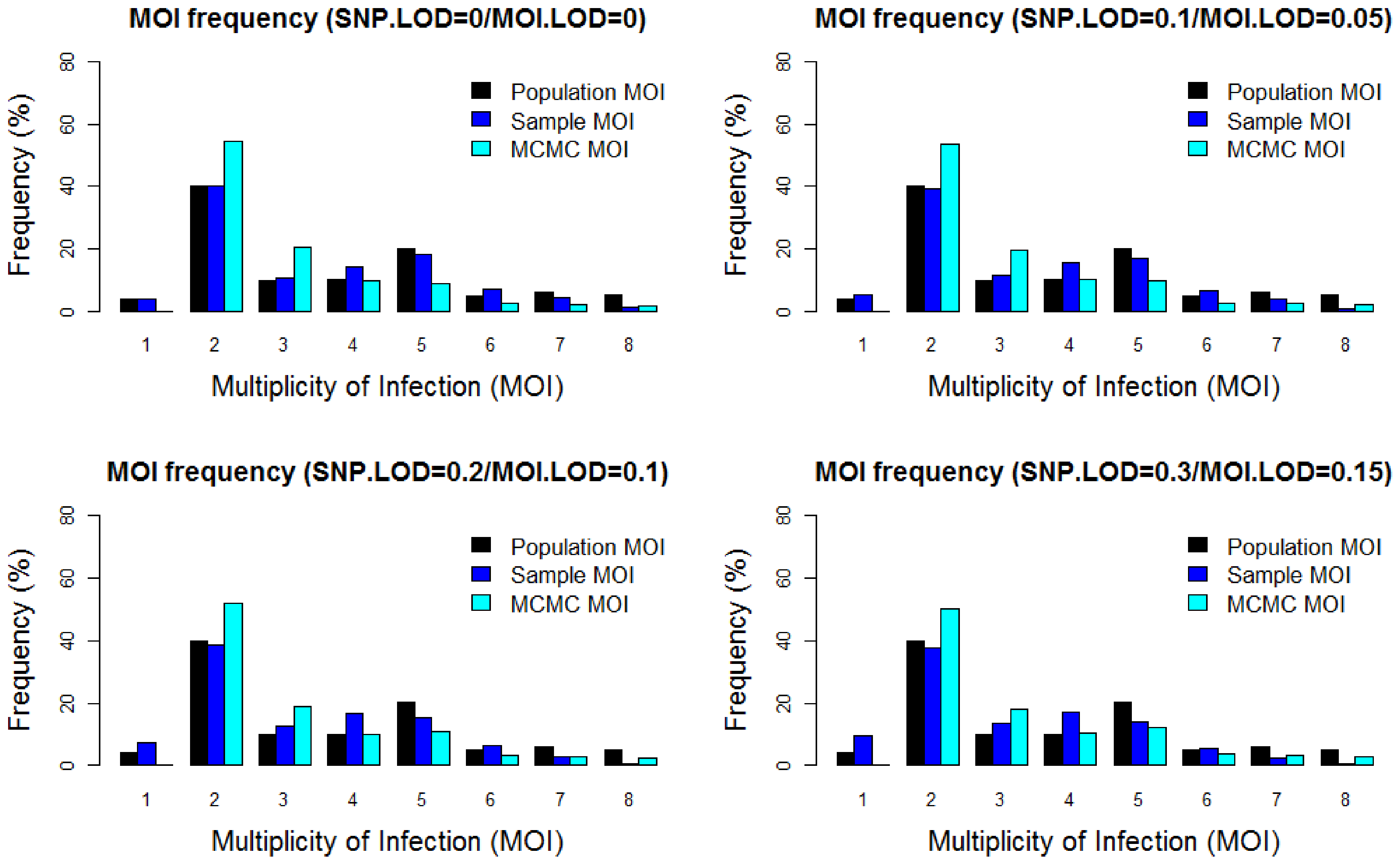

Figure S18: The MOI frequency distributions in the whole patient population ("Population MOI"), the patient population in the sample under analysis ("Sample MOI") and the distribution estimated by the MCMC algorithm (with change MOI) allowing for different levels of genetic detection i.e. LoD_{SNP}=0%/10%/20%/30% and LoD_{MOI}=0%/5%/10%/15%. This came from algorithm 4 (i.e. start with Jaki et al distribution) mean after 100 iterations.

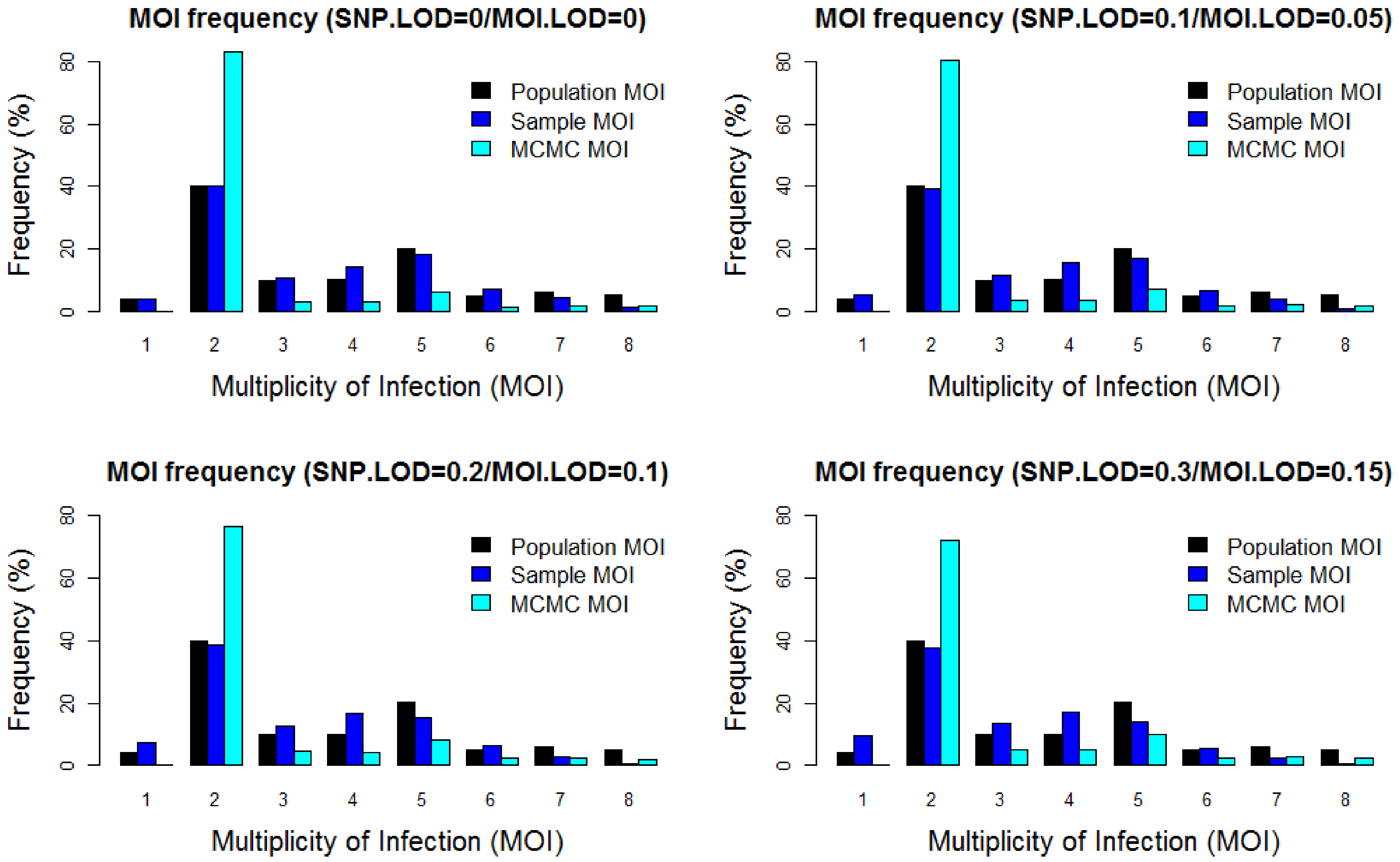

Figure S19: The correlation (R^2) between population/sample and estimated haplotype frequency among 3 SNPs across statistical methods and LoD_{SNP}=0.00, LoD_{MOI}=0.00, the line through [0,1] represents the situation where both estimates are identical.

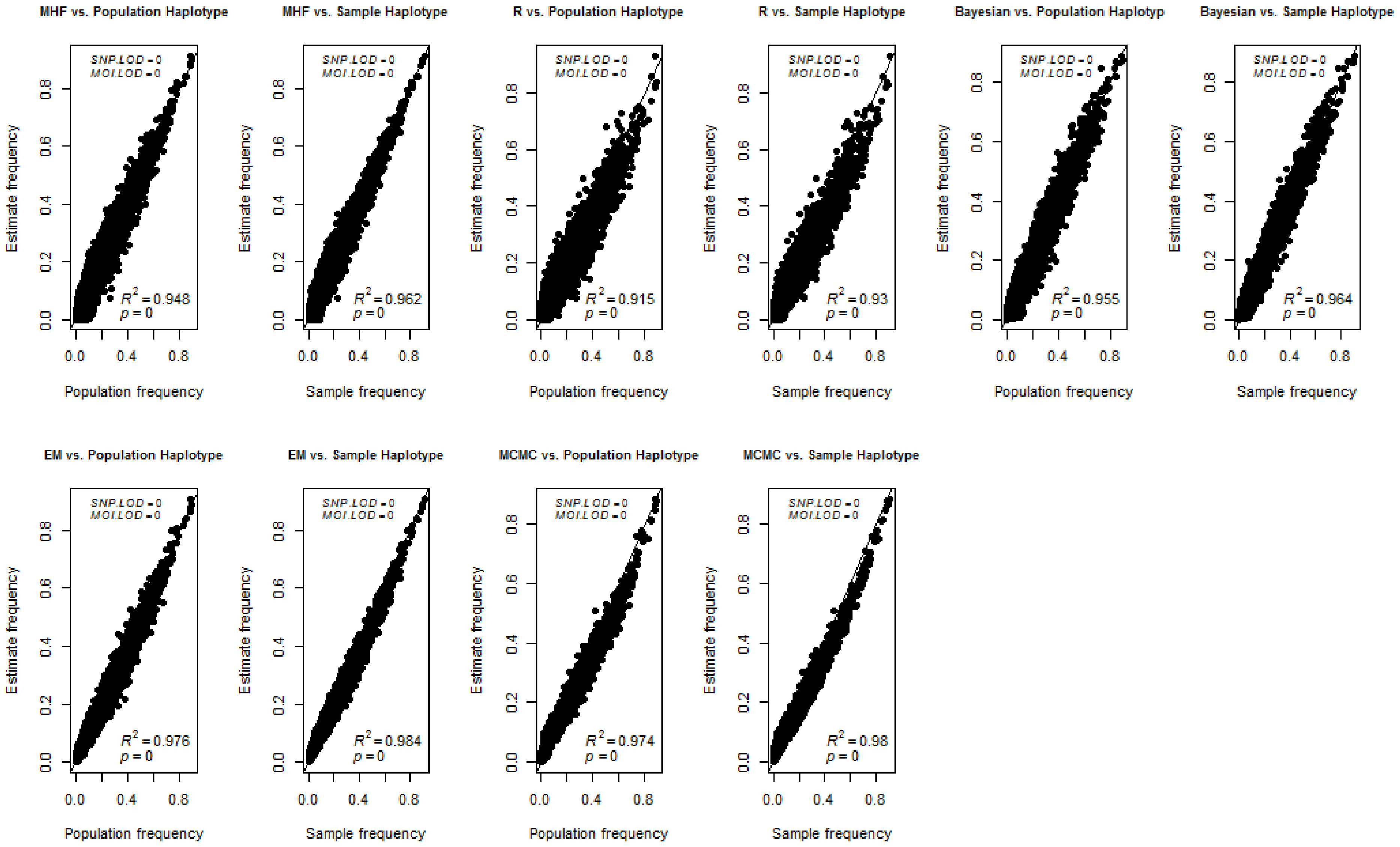

Figure S20: The correlation (R^2) between population/sample and estimated haplotype frequency among 3 SNPs across statistical methods and LoD_{SNP}=0.30, LoD_{MOI}=0.15, the line through [0,1] represents the situation where both estimates are identical.

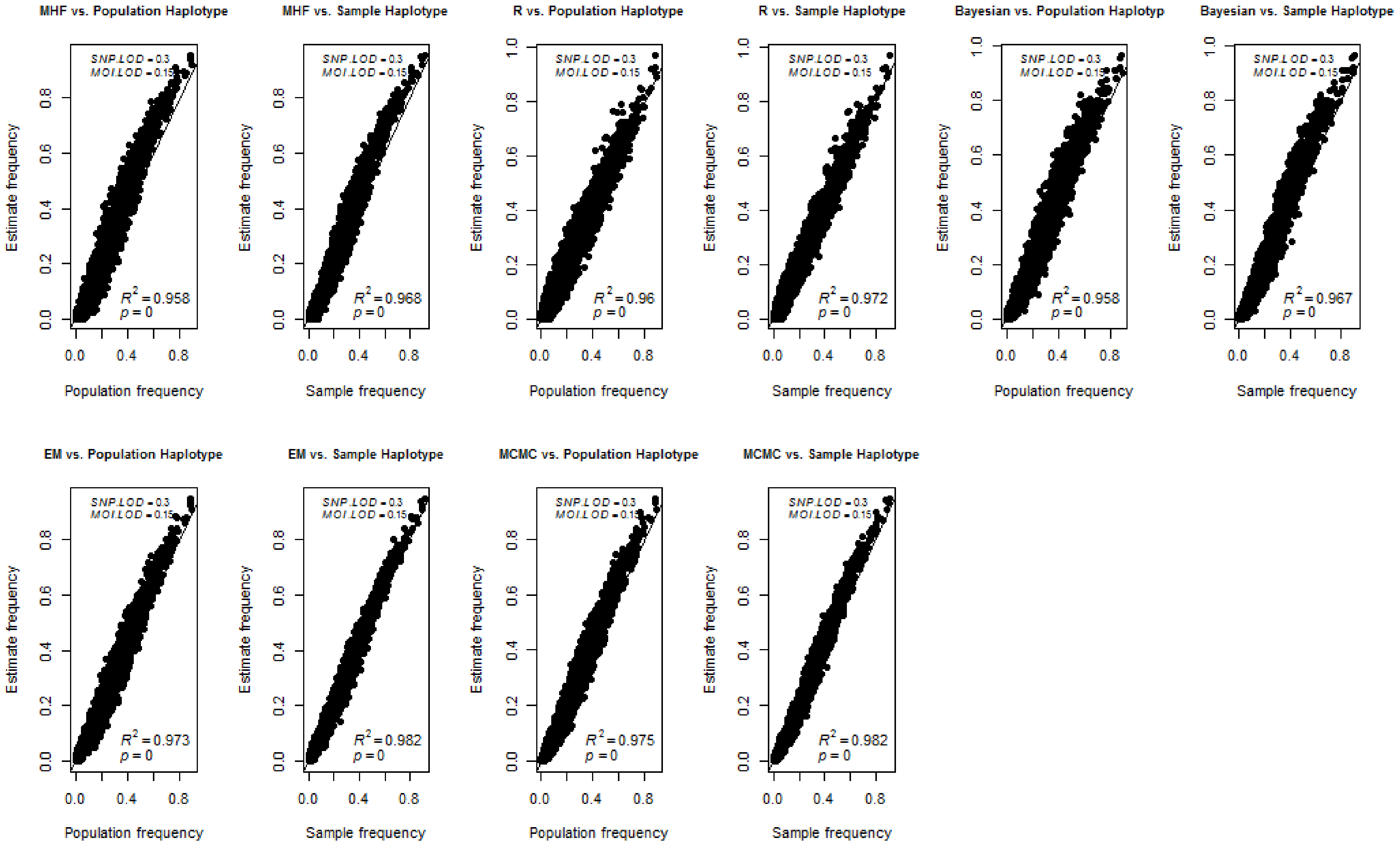

Figure S21: The change coefficient (*C*) between population/sample and estimated haplotype frequency across statistical methods among 3SNPs and LoD_{SNP}=0.00, LoD_{MOI}=0.00.

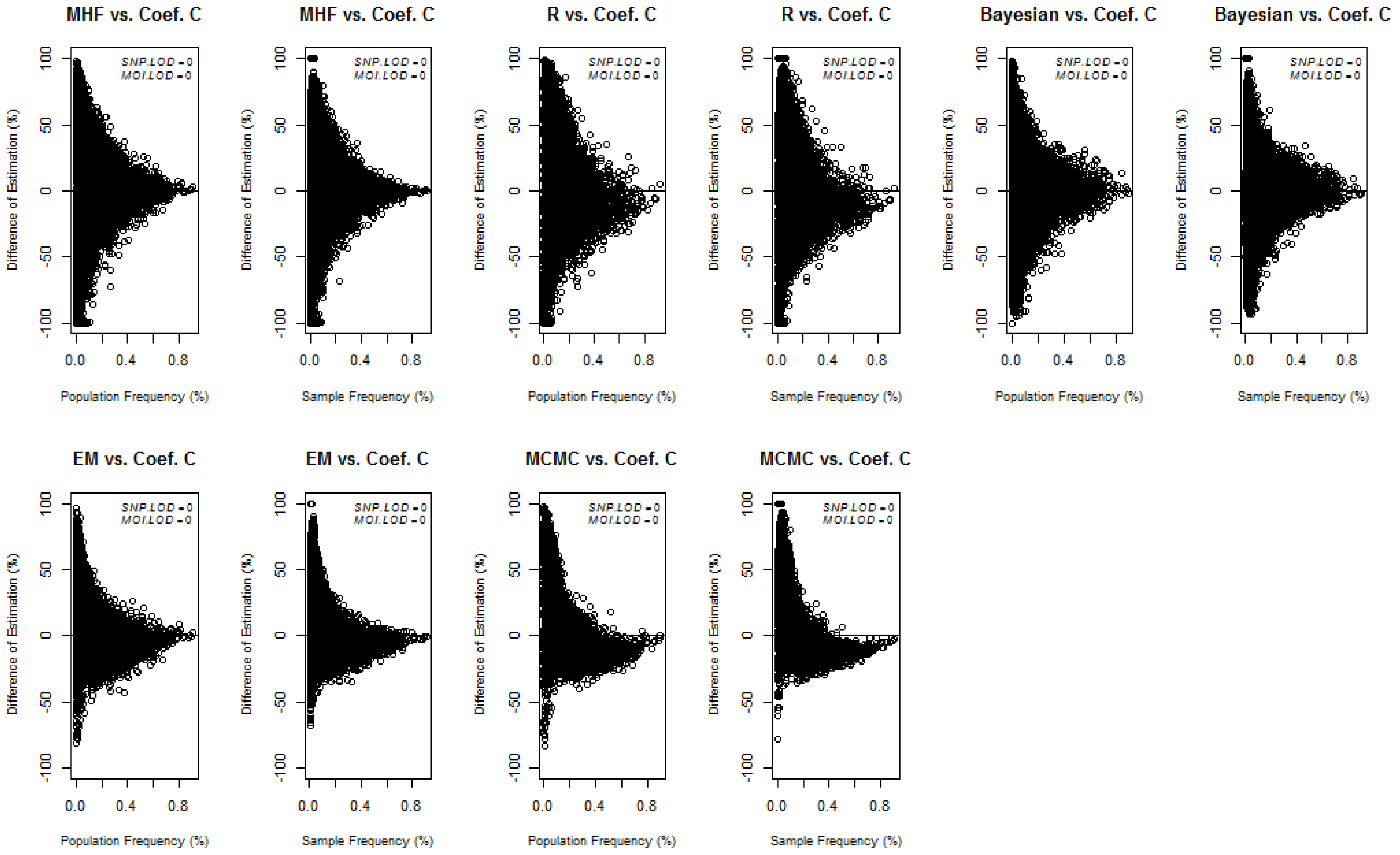

Figure S22: The change coefficient (*C*) between population/sample and estimated haplotype frequency across statistical methods among 3SNPs and LoD_{SNP}=0.30, LoD_{MOI}=0.15.

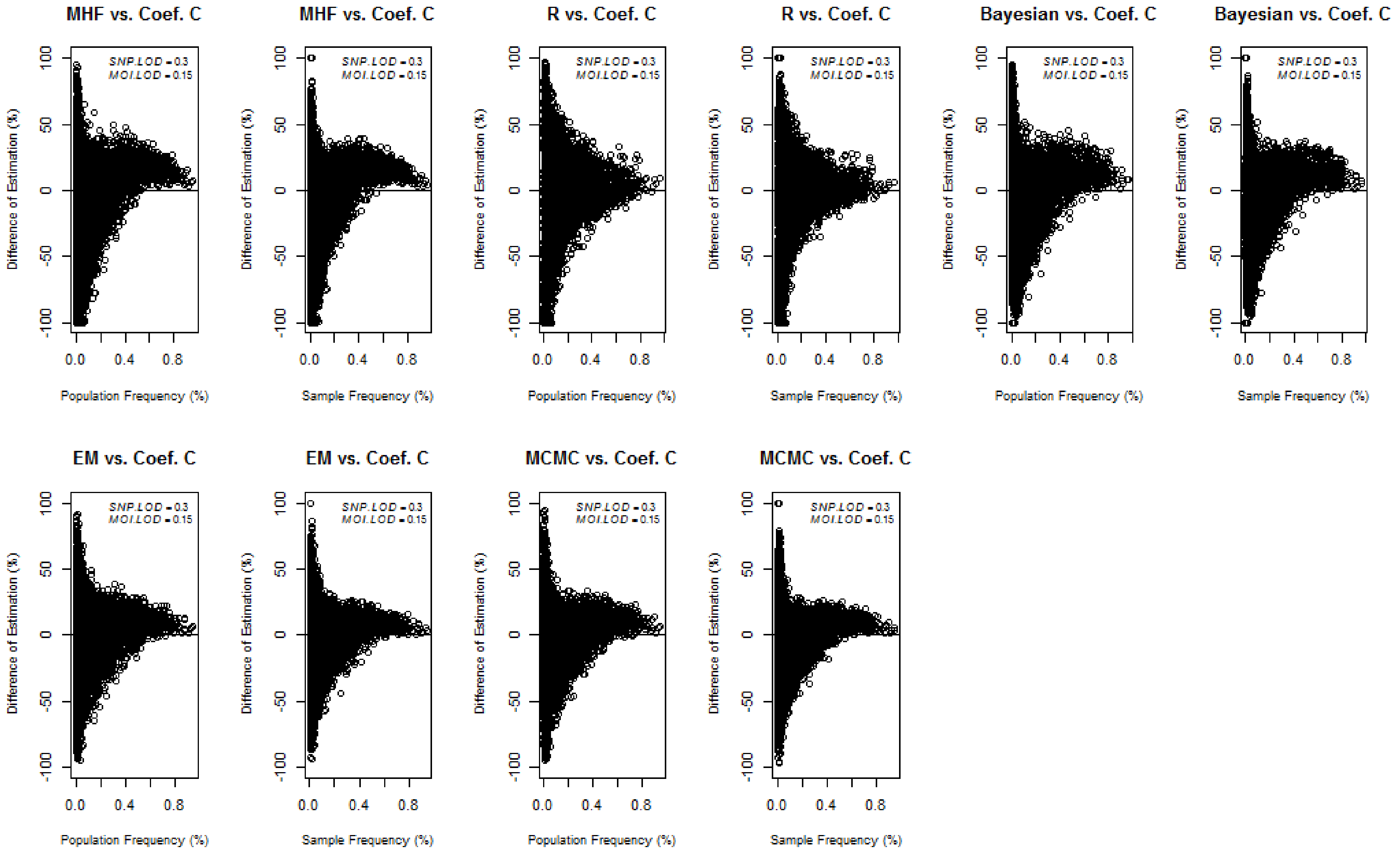

Figure S23: The similarity index (*I_F*) and mean squared error (*MSE*) of the estimates haplotype frequency compared population/sample haplotype frequency across statistical methods (MHF, MalHaploFreq; R-EM, malaria em; Bayesian, Bayesian statistic; EM, EM algorithm; MCMC, Markov Chain Monte Carlo) among 3 SNPs and four conditions of LoD_{SNP} (30%, 20%, 10%, 0%) and LoD_{MOI} (15%, 10%, 5%, 0%).

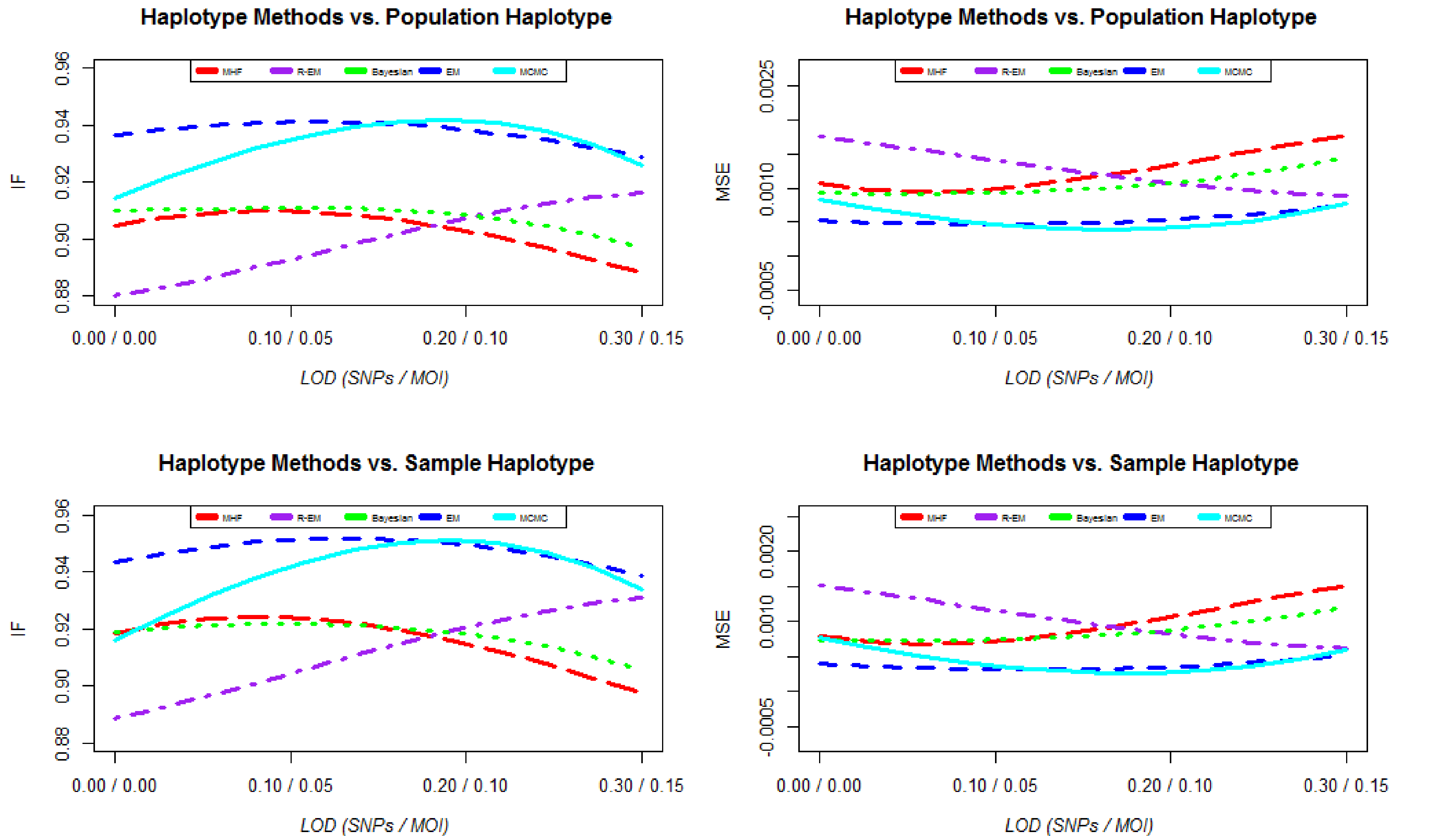

Figure S24: A trace plot and density plots of the iteration number against the value of the haplotype frequencies at 100 iterations.

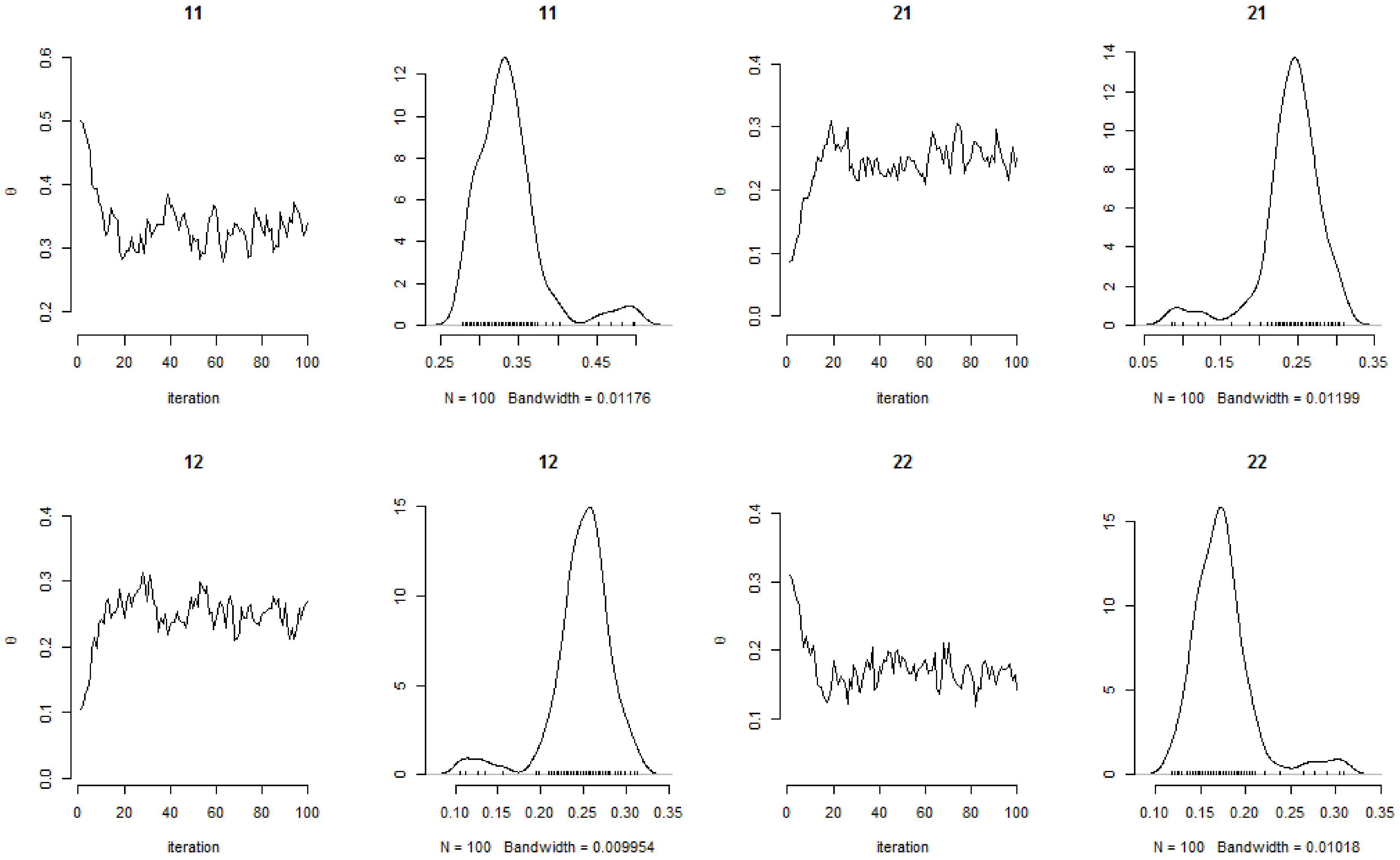

Figure S25: Autocorrelation plots to assess the autocorrelations between the haplotype frequencies of markov chain at 100 iterations.

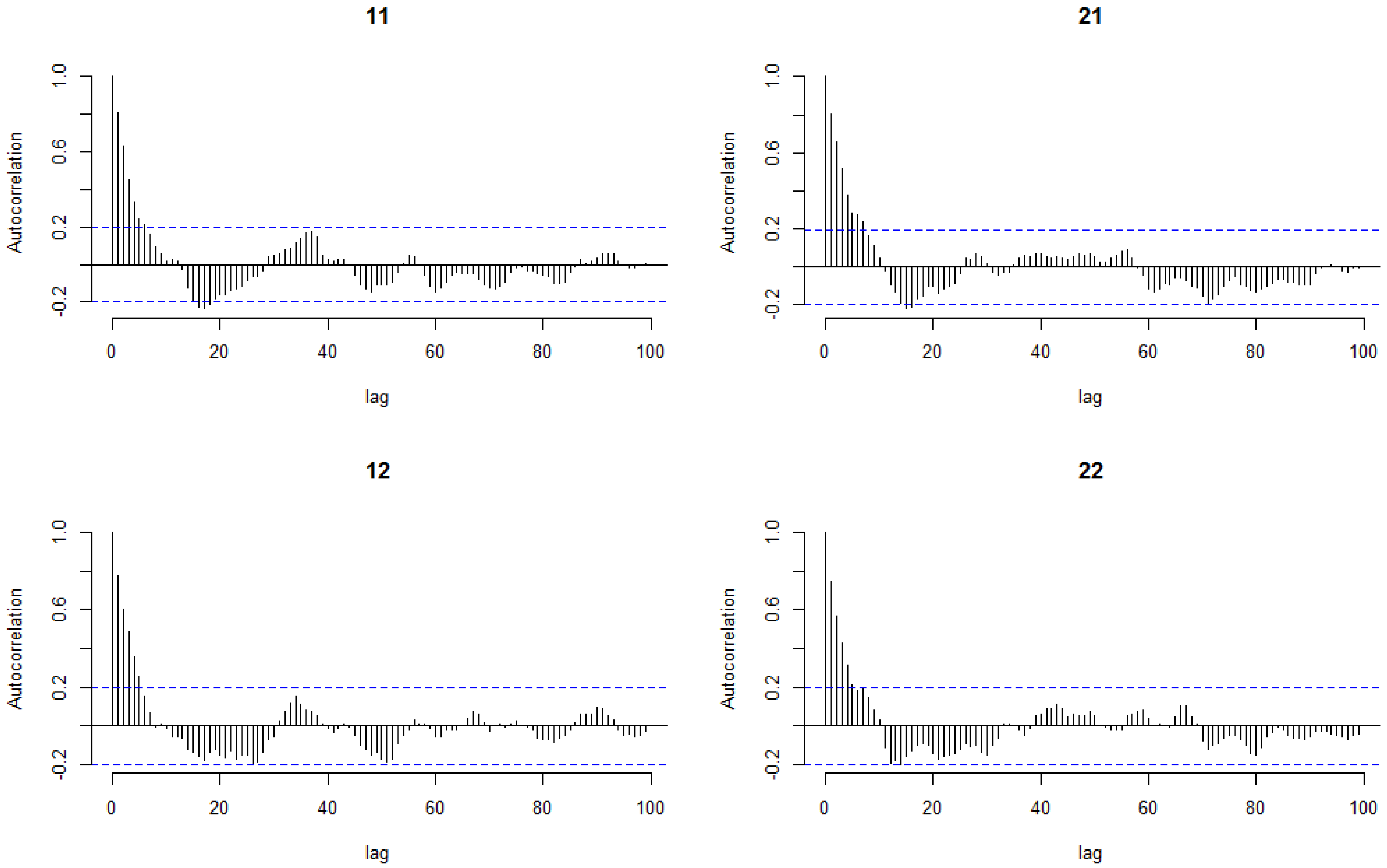

Figure S26: Gelman Rubin Brooks plot (shrink factor as the number of iterations) and mean plot (mean as the number of iterations) with 2 chains at 100 iterations.

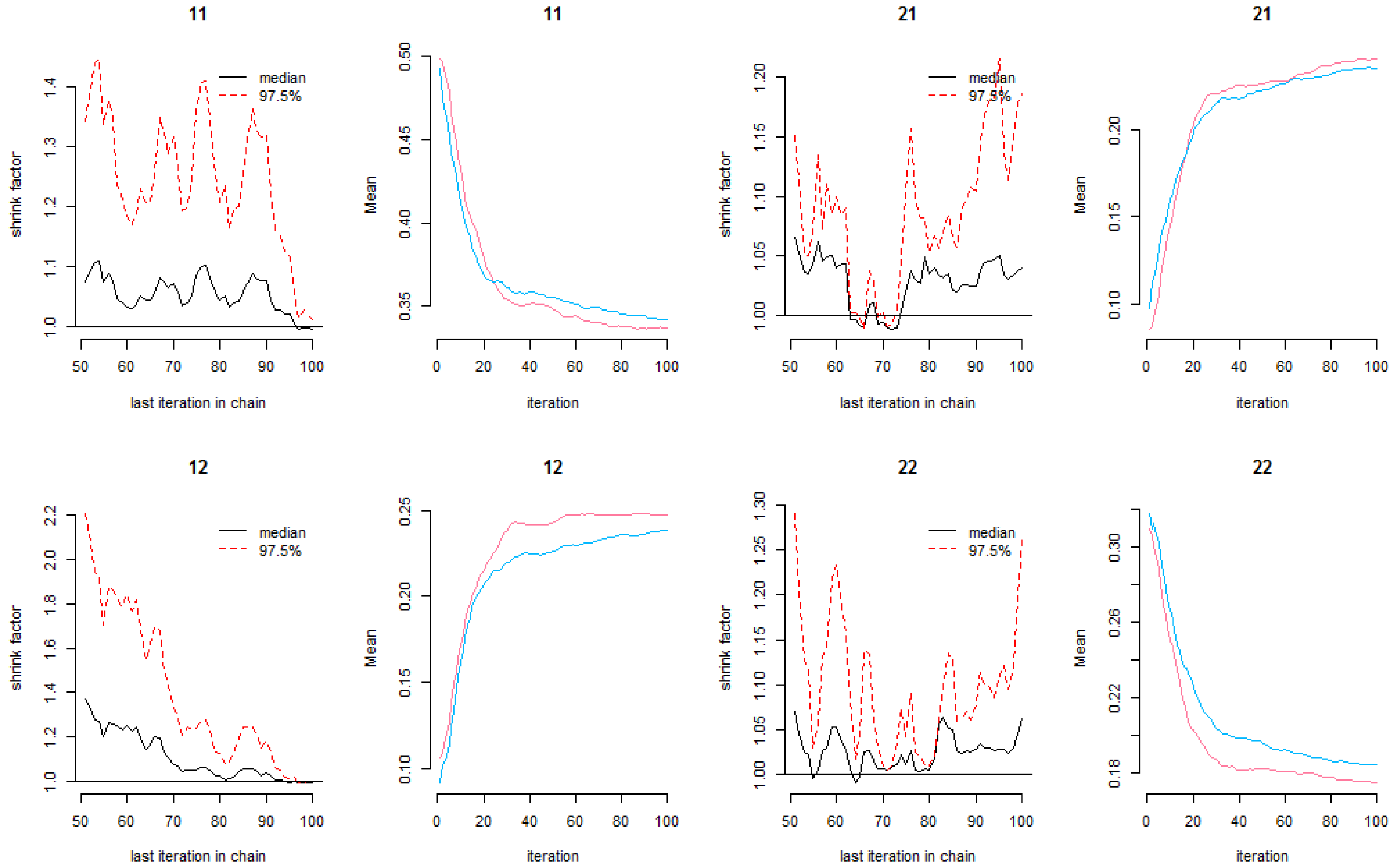

Figure S27: A trace plot and density plots of the iteration number against the value of the haplotype frequencies at 1000 iterations.

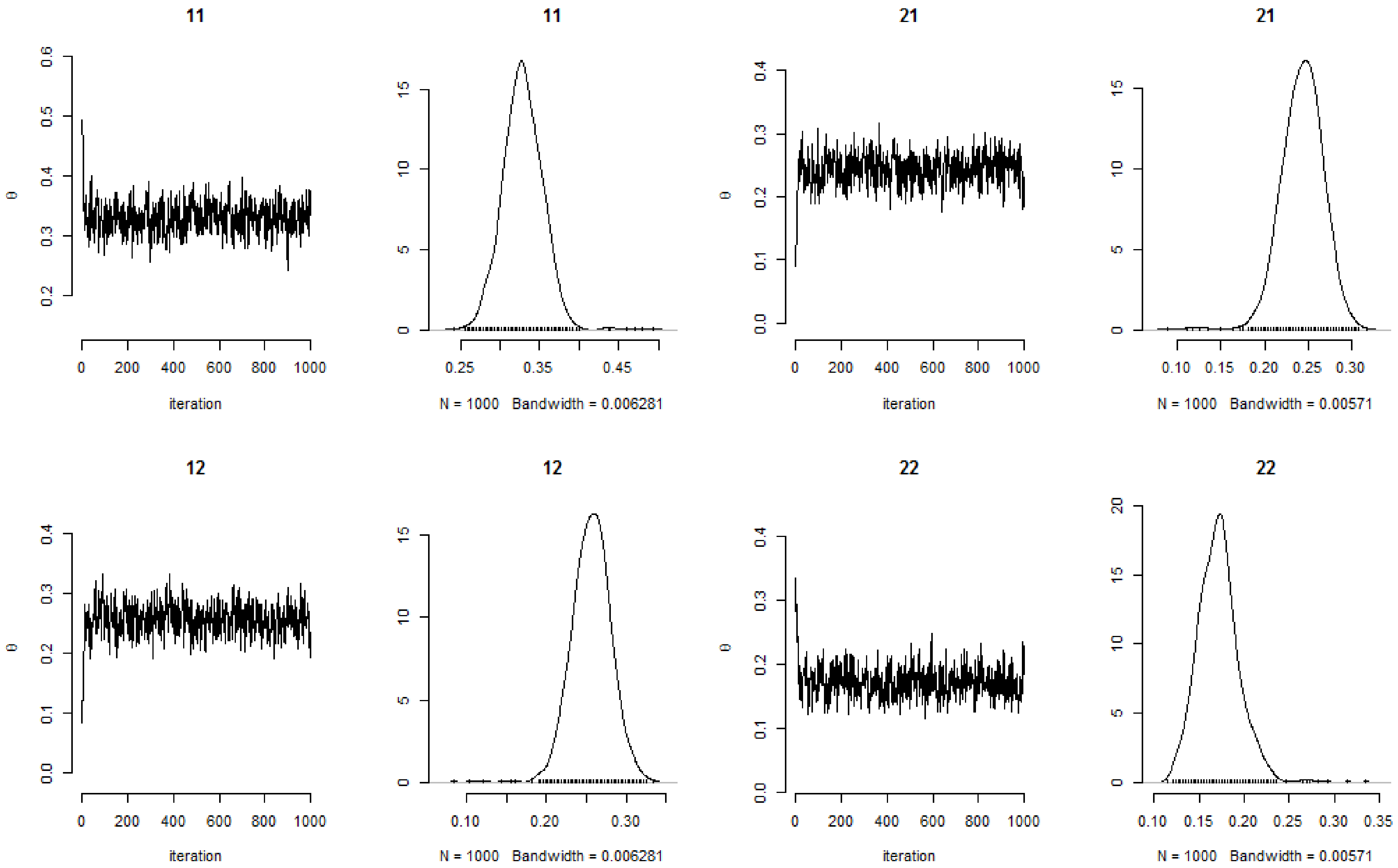

Figure S28: Autocorrelation plots to assess the autocorrelations between the haplotype frequencies of markov chain at 1000 iterations.

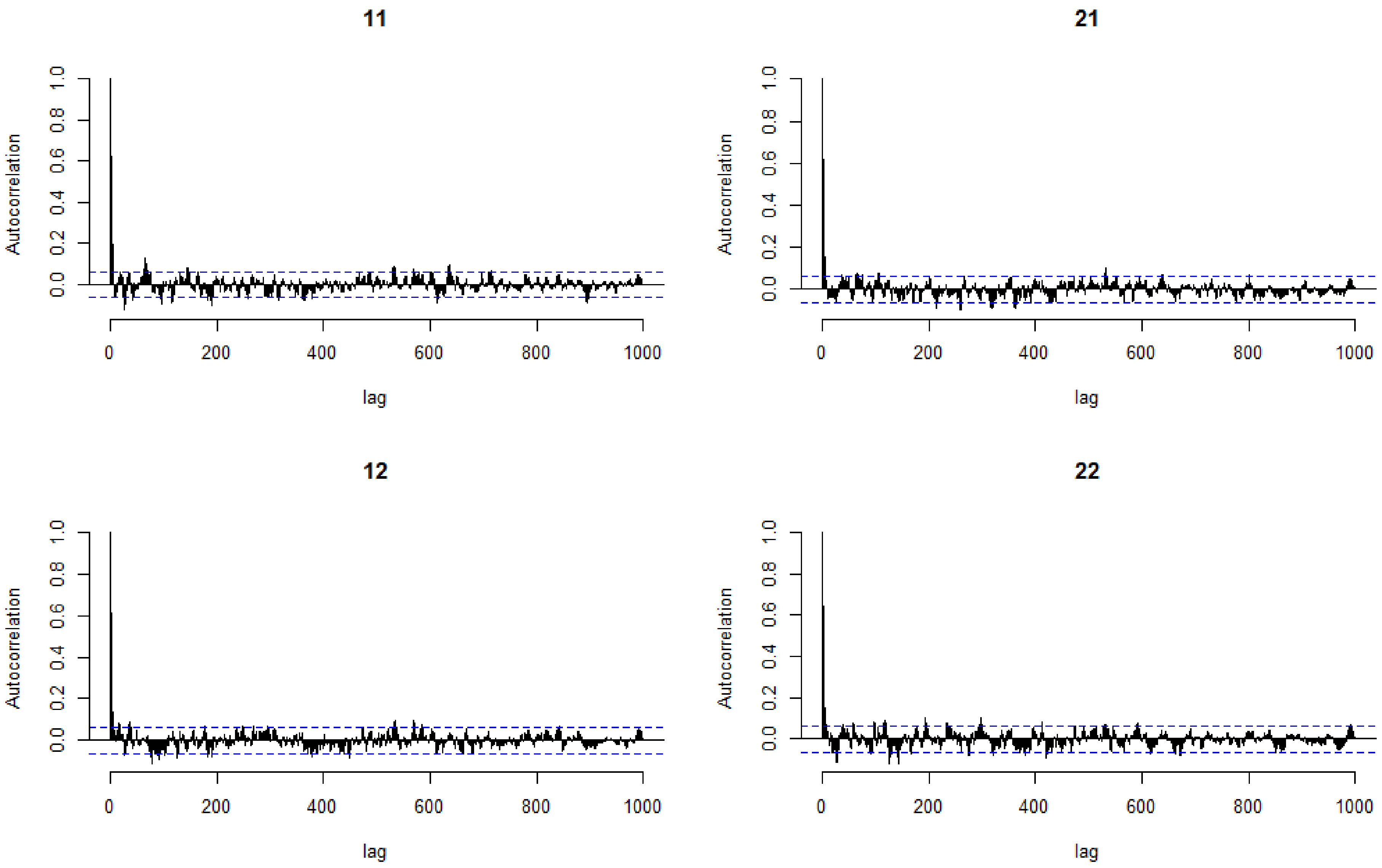

Figure S29: Gelman Rubin Brooks plot (shrink factor as the number of iterations) and mean plot (mean as the number of iterations) with 2 chains at 1000 iterations.

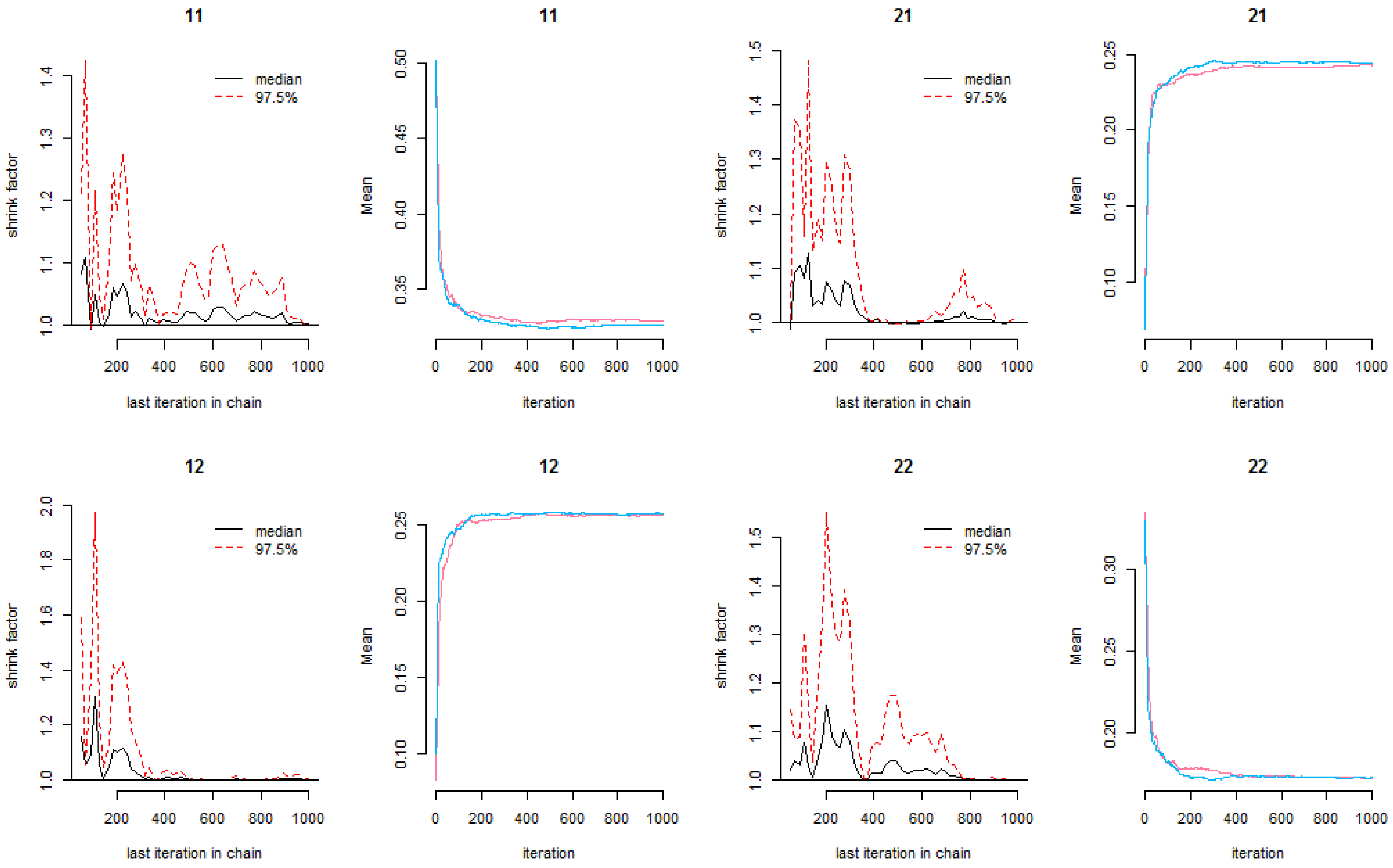

Supplement: Supplementary file 1 — 10.1186/s12936-016-1473-5 Data that support and expand some of the interpretations and conclusions drawn in the main text, but whose inclusion would detract from the main argument. [file 12936_2016_1473_MOESM1_ESM.pdf]
